# Mutual regulation between phosphofructokinase 1 platelet isoform and VEGF promotes glioblastoma tumor growth

**DOI:** 10.1038/s41419-022-05449-6

**Published:** 2022-11-26

**Authors:** Je Sun Lim, YuJie Shi, Su Hwan Park, So Mi Jeon, Chuanbao Zhang, Yun-Yong Park, Rui Liu, Jing Li, Wan-Seob Cho, Linyong Du, Jong-Ho Lee

**Affiliations:** 1 Department of Health Sciences, The Graduate School of Dong-A University, Busan, 49315 Republic of Korea; 2 State Key Laboratory of Oral Diseases, National Clinical Research Center for Oral Diseases, Chinese Academy of Medical Sciences Research Unit of Oral Carcinogenesis and Management, West China Hospital of Stomatology, Sichuan University, Chengdu, Sichuan 610041 P.R. China; 3 Department of Neurosurgery, Beijing Tiantan Hospital, Capital Medical University, Beijing, 100070 P.R. China; 4 Department of life Science, Chung-Ang University, Seoul, 06974 Republic of Korea; 5 Key Laboratory of Laboratory of Medicine, Ministry of Education of China, School of Laboratory Medicine and Life Science, Wenzhou Medical University, Wenzhou, Zhejiang 325000 P.R. China; 6 Department of Biomedical Sciences, Dong-A University, Busan, 49315 Republic of Korea

**Keywords:** Cancer metabolism, Tumour angiogenesis

## Abstract

Glioblastoma (GBM) is a highly vascular malignant brain tumor that overexpresses vascular endothelial growth factor (VEGF) and phosphofructokinase 1 platelet isoform (PFKP), which catalyzes a rate-limiting reaction in glycolysis. However, whether PFKP and VEGF are reciprocally regulated during GBM tumor growth remains unknown. Here, we show that PFKP can promote EGFR activation-induced VEGF expression in HIF-1α-dependent and -independent manners in GBM cells. Importantly, we demonstrate that EGFR-phosphorylated PFKP Y64 has critical roles in both AKT/SP1-mediated transcriptional expression of *HIF-1α* and in the AKT-mediated β-catenin S552 phosphorylation, to fully enhance *VEGF* transcription, subsequently promoting blood vessel formation and brain tumor growth. Levels of PFKP Y64 phosphorylation in human GBM specimens are positively correlated with HIF-1α expression, β-catenin S552 phosphorylation, and VEGF expression. Conversely, VEGF upregulates PFKP expression in a PFKP S386 phosphorylation-dependent manner, leading to increased PFK enzyme activity, aerobic glycolysis, and proliferation in GBM cells. These findings highlight a novel mechanism underlying the mutual regulation that occurs between PFKP and VEGF for promoting GBM tumor growth and also suggest that targeting the PFKP/VEGF regulatory loop might show therapeutic potential for treating GBM patients.

## Introduction

Angiogenesis is indispensable for both physiologic and pathologic processes [1, 2]. Vascular endothelial growth factor (VEGF) is a critical regulator of both physiologic and pathologic processes [1, 2]. In particular, tumor growth and metastasis highly depend on angiogenesis because the formation of new blood vessels is required for the continued growth and malignant dissemination of solid tumors [1, 2]. Consequently, excessive angiogenesis is often observed in the pathogenesis of most tumors [2]. Glioblastoma (GBM) is the most frequent primary adult brain tumor with highly developed abnormal vascular structures and VEGF overexpression [3]. Defining the mechanisms that regulate VEGF expression in GBM cells has important implications for understanding tumor progression, thereby providing clinically relevant information that might suggest strategies for blocking angiogenesis in the pathogenesis of GBM tumors.

Hypoxia is a common feature of the microenvironment of solid tumors [4]. It is well-known that hypoxia upregulates VEGF via the transcription factor hypoxia-inducible factor-1 (HIF-1) [5, 6]. HIF-1, which is a heterodimeric protein consisting of an α subunit that is induced by hypoxia and a β subunit that is constitutively present [6], binds to a specific consensus sequence within the hypoxia response element (HRE) in the VEGF promoter [7]. However, many cancer cells, including GBM cells, express high levels of VEGF, even under normoxic conditions [8–12], suggesting that intrinsic factors can lead to VEGF upregulation independent of the environment. Epidermal growth factor receptor (EGFR) is overexpressed or mutated in many types of cancers, including GBM [13], and correlates with poor clinical prognosis [14]. Although EGFR activation has been shown to upregulate VEGF expression in many types of cancers [9, 10, 12], the specific mechanisms involved in such upregulation remain to be elucidated.

Metabolic reprogramming is an emerging hallmark of cancer [2]. Metabolic reprogramming and other cellular activities are essential for biological processes and are important for tumor development [15]. Beside their canonical metabolic functions, metabolic enzymes possess nonmetabolic functions in cancer cells, which play crucial roles in tumorigenesis [15]. In the glycolytic pathway, phosphofructokinase 1 (PFK1) catalyzes the conversion of fructose 6-phosphate and ATP to fructose-1,6-bisphosphate and ADP, one of the key regulatory and rate-limiting steps of glycolysis [16]. PFK1 exists in multiple tetrameric isozymic forms, including a platelet isoform (PFKP), a liver isoform (PFKL), and a muscle isoform (PFKM). The expression and composition of isoforms can vary depending on the tissue and cell type [16, 17]. We have previously reported that all three isoforms are expressed in GBM cells, with PFKP being the prominent PFK1 isoform in GBM cells, and PFKP is overexpressed in human GBM specimens [18]. Interestingly, we have also found that PFKP has a nonmetabolic function; PFKP binds to EGFR upon EGFR activation, leading to EGFR-mediated phosphorylation of PFKP Y64, which in turn binds to an SH2 domain of the p85 subunit of phosphoinositide 3-kinase (PI3K) to activate PI3K. The PFKP Y64 phosphorylation-dependent activation of PI3K/AKT enhances aerobic glycolysis in brain tumorigenesis [19]. However, whether PFKP has a critical role in VEGF expression-induced angiogenesis and vice versa during GBM development remains unknown.

In this study, we demonstrate that PFKP promotes EGFR activation-induced VEGF expression in HIF-1α-dependent and -independent manners in GBM cells, leading to enhanced blood vessel formation and brain tumor growth. Conversely, VEGF upregulates PFKP expression, thereby enhancing PFK enzyme activity, aerobic glycolysis, and proliferation in GBM cells.

## Materials and methods

### Materials

Rabbit polyclonal antibodies recognizing PFKP (12746; 1:1000 for immunoblotting), β-catenin (pS552, 9566; 1:1000 for immunoblotting, 1:300 for immunohistochemistry), EKR1/2 (pT202/pY204, 9101; 1:1000 for immunoblotting), ERK1/2 (9102; 1:1000 for immunoblotting), AKT (pS473, 4060; 1:1000 for immunoblotting), AKT (9272; 1:1000 for immunoblotting), p38 (pT180/pY182, 9211; 1:1000 for immunoblotting), p38 (9212; 1:1000 for immunoblotting), JNK (pT183/pY185, 9251; 1:1000 for immunoblotting), c-Jun (pS63, 2361; 1:1000 for immunoblotting), c-Jun (9165; 1:1000 for immunoblotting), IκBα (pS32/36, 9246; 1:1000 for immunoblotting), IκBα (9242; 1:1000 for immunoblotting), p65 (pS536, 3033; 1:1,000 for immunoblotting), p65 (8242; 1:1,000 for immunoblotting), PGK1 (68540; 1:1000 for immunoblotting), PKM2 (3198; 1:1000 for immunoblotting), and c-Myc (E5Q6W, 18583; 1:1000 for immunoblotting) were purchased from Cell Signaling Technology (Danvers, MA). Mouse monoclonal antibodies for VEGF (C-1, sc-7269; 1:500 for immunoblotting, 1:100 for immunohistochemistry), SP1 (1C6, sc-420; 1:500 for immunoblotting), PFKL (A-6, sc-393713; 1:500 for immunoblotting), PFKM (sc-67028; 1:1000 for immunoblotting), HK2 (sc-130858; 1:500 for immunoblotting), LDHA (sc-137243; 1:500 for immunoblotting), GAPDH (sc-47724; 1:500 for immunoblotting), PFK2 (sc-377416; 1:500 for immunoblotting), enolase (sc-271384; 1:500 for immunoblotting), aldolase (sc-390733; 1:500 for immunoblotting), cyclin D1 (A-12, sc-8396; 1:500 for immunoblotting), and mithramycin A (SC-200909) were purchased from Santa Cruz Biotechnology (Santa Cruz, CA). Rabbit polyclonal EGFR antibody (pY869, 11229; 1:1000 for immunoblotting) was purchased, and rabbit polyclonal PFKP antibody (pY64; 5 mg/mL used for IHC staining) [19] were obtained from Signalway Antibody (College Park, MD). Mouse monoclonal antibody PGAM1 (GTX629754, 1:500 for immunoblotting) was purchased from GeneTex (Irvine, CA). Rabbit polyclonal antibody that recognizes PFKP (pS386; 1:1,000 for immunoblotting) [18] was obtained from Signalway Biotechnology (Pearland, TX). Rabbit monoclonal antibody for HIF-1α (EP1215Y, ab51608; 1:1000 for immunoblotting, 1:300 for immunohistochemistry) and rabbit polyclonal antibody for CD31 (ab28364; 1:300 for immunohistochemistry) were purchased from Abcam (Cambridge, MA). Mouse monoclonal antibody for EGFR (610016; 1:1000 for immunoblotting) was purchased from BD Biosciences (San Jose, CA). Mouse monoclonal antibodies for FLAG (clone M2, F3165; 1:5000 for immunoblotting), HA (H6908; 1:5000 for immunoblotting), tubulin (clone B-5-1-2, T6074; 1:5000 for immunoblotting), human recombinant EGF (E9644), hygromycin B (H3274), puromycin (P8833), cycloheximide (66-81-9), and actinomycin D (A1410) were purchased from Sigma (St. Louis, MO). Goat anti-mouse IgG (HRP; RSA1122) and goat anti-rabbit IgG (HRP; RSA1221) secondary antibodies were purchased from BioActs (Republic of Korea). G418 (4727878001) was purchased from Roche (Basel, Switzerland). LY294002 (L-7988), SP600125 (S-7979), PD98059 (P-4313), and SB203580 (S-3400) were purchased from LC Laboratories (Woburn, MA). NF-κB inhibitor (481412) was purchased from EMD Biosciences (San Diego, CA). MK-2206 (S1078) and HIF-1α inhibitor (S7612) were purchased from Selleck Chemicals (Houston, TX). Recombinant human VEGF165 (100-20) was purchased from Peprotech Korea (Seoul, Republic of Korea).

### Cell culture

U373MG GBM cells were purchased from the Korean Cell Line Bank (KCLB; Seoul, Republic of Korea). U251, LN18, T98G, A172, and LN229 GBM cells were kindly provided by Dr. Hyunggee Kim (Korea University, Seoul, Republic of Korea). All cells were routinely tested for mycoplasma. These GBM cell lines were maintained in Dulbecco’s modified Eagle’s medium (DMEM) supplemented with 10% fetal bovine serum (Capricorn Scientific, Germany). GSCs (XO6) originally isolated from human GBM specimens of patients undergoing surgery [20, 21] have been studied by several other groups [22, 23]. They were kindly provided by Dr. Yong Tae Kwon’s group (Seoul National University, Seoul, Republic of Korea). GSCs were maintained in Neurobasal Plus Medium (ThermoFisher; Pittsburgh, PA) supplemented with 2% B-27 (minus vitamin A; ThermoFisher, Pittsburgh, PA), EGF (20 ng/mL), and FGF (20 ng/mL) (PeproTech, Seoul, Republic of Korea).

### Transfection

Cells were plated at a density of 4 × 10^5^ cells per 60-mm dish or 1 × 10^5^ cells per well of a 6-well plate at 18 h before transfection. Transfection was performed using Lipofectamine^2000^ transfection reagent (ThermoFisher Scientific; Pittsburgh, PA) according to the manufacturer’s instructions. SP1 siRNA (116546) was purchased from ThermoFisher Scientific (Pittsburgh, PA). Transfection of SP1 siRNA was performed using Lipofectamine^TM^ RNAiMAX transfection reagent (ThermoFisher; Pittsburgh, PA) according to the manufacturer’s instructions.

### DNA constructs and mutagenesis

Polymerase chain reaction (PCR)-amplified human PFKP, HIF-1α, and β-catenin were cloned into pcDNA3.1/hygro(+)-Flag vector. pcDNA3.1/hygro(+)-Flag PFKP Y64F, Flag PFKP S386A, or β-catenin S552A was created using the QuikChange site-directed mutagenesis kit (Stratagene, La Jolla, CA). pLV/β-catenin deltaN90 (CA β-catenin) and pECE-Myr-HA-AKT1(delta4-129) were purchased from Addgene (Cambridge, MA). shRNA-resistant (r) PFKP contained a448c, g450c, c453t, and c456g mutations. The following pGIPZ shRNAs were used: control shRNA oligonucleotide, GCTTCTAACACCGGAGGTCTT; human PFKP shRNA#1 oligonucleotide, AGGAACGGCCAGATCGATA; human PFKP shRNA#2 oligonucleotide, TGGAGTGGATCACTGCAAA; and human β-catenin shRNA oligonucleotide, TTACCACTCAGA GAAGGAG.

### Quantitative real-time PCR analysis

Total RNAs were prepared from tumor cells using an RNeasy Mini kit (Qiagen; Valencia, CA) according to the manufacturer’s instructions. For synthesizing cDNAs, 2 μg of total RNAs and reverse transcriptase (Superscript II Preamplification System, Gibco-BRL; Gaithersburg, MD) were used. Real-time PCR was performed on an ABI Prism 7500 sequence detection system using an SYBR® Green PCR Master Mix (Applied Biosystems; Foster City, CA) and following the manufacturer’s protocols. The ABI 7500 sequence detector was programmed with the following PCR conditions: 40 cycles of 15-s denaturation at 95 °C and 1-minute amplification at 60 °C. All reactions were run in triplicate and normalized to the housekeeping gene *HPRT*. Relative expression levels were calculated using the comparative cycle threshold (CT) method. The following primer pairs were used for quantitative real-time PCR: human *HIF-1α*, 5′-CATAAAGTCTGCAACATGGAAGGT-3′ (forward), and 5′-ATTTAGTGGGTGAGGAATGGGTT-3′ (reverse); human *VEGF*, 5′-TGCAGATTATGCGGATCAAACC-3′ (forward) and 5′-TGCATTCACATTTGTTGTGCTGTAG-3′ (reverse); and human *HPRT*, 5′-CATTATGCTGAGGATTTGGAAAGG-3′ (forward) and 5′-CTTGAGCACACAGAGGGCTACA-3′ (reverse).

### Western blot analysis

Proteins were extracted from cultured cells using a cell lysis buffer (50 mM Tris-HCl, [pH 7.5], 0.1% sodium dodecyl sulfate, 1% Triton X-100, 150 mM NaCl, 1 mM DTT, 0.5 mM EDTA, 100 µM sodium orthovanadate, 100 µM sodium pyrophosphate, 1 mM sodium fluoride, and proteinase inhibitor cocktail). Cell extracts were clarified via centrifugation at 13,400*g*, and the supernatants (1.5 mg protein/mL) were subjected to immunoblot analysis with corresponding antibodies. Band intensity was quantified using ImageJ 1.53e software (National Institutes of Health). Each experiment was repeated at least three times. Full scans of original immunoblots are displayed in Supplementary Figure 6.

### Luciferase reporter assay

The tumor cells were co-transfected with pGL3 empty vector, a pGL3-HRE-luciferase plasmid containing five copies of HREs from the human VEGF gene, pGreenFire1-mCMV, or pGreenFire1-SP1 (System Biosciences; Palo Alto, CA) and pRL-TK vector (as an inner control that contained Renilla luciferase sequences (Promega; Madison, WI)) using Lipofectamine^2000^ transfection reagent (ThermoFisher Scientific; Pittsburgh, PA) according to the manufacturer’s instructions, and then grown under different experimental conditions. After incubation, firefly and Renilla luciferase activities were measured using a Dual-Luciferase® Reporter Assay System (Promega; Madison, WI). The ratio of firefly/Renilla luciferase was then determined.

### Chromatin immunoprecipitation (ChIP) assay

A ChIP assay was performed using a SimpleChIP Enzymatic Chromatin IP kit (9003; Cell Signaling Technology). Chromatin prepared from 2 × 10^6^ cells (in a 10-cm dish) was used to determine the total DNA input and then incubated overnight with SP1 antibody or with normal mouse IgG at 4 °C overnight. Immunoprecipitated chromatin was detected using real-time PCR. The PCR primer sequences for the *HIF-1α* promoter were SP1 #P1, 5′-CGAGGCGAAGTCTGCTTTTT-3′ (forward) and 5′-TCCTACTCTTGGTGCAGTAATG-3′ (reverse); SP1 #P2, 5′-TCGCTCGCCATTGGATCTCG-3′ (forward) and 5′-GCGCGCGGGGAGGGGAGAGG-3′ (reverse); and SP1 #P3, 5′-CCCCCTCTCCCCTCCCCGCG-3′ (forward) and 5′-GAGGAGCTGAGGCAGCGTCA-3′ (reverse).

### Tube formation assay

Human umbilical vein endothelial cells (HUVECs) were maintained in endothelial cell growth basal medium-2 (EBM-2; Lonza, Walkersville, MD) supplemented with SingleQuots^TM^ Supplements and Growth Factors (Lonza; USA, MD). Matrigel (Corning, Flintshire, UK) was diluted with serum-free EBM-2 medium and used to coat in 96-well plates at 37 °C for 1 h. HUVECs were seeded at a density of 1 × 10^4^ cells per well of a 96-well plate in EBM-2 or conditional medium. The number of tube formations was imaged and analyzed using phase contrast microscopy and ImageJ 1.53e software (National Institutes of Health).

### Measurement of glucose consumption and lactate production

Cells were seeded into culture dishes, and the medium was changed to non-serum-containing DMEM after 12 h. The culture medium was collected at indicated time points to measure glucose and lactate concentrations. Glucose levels were determined using a glucose (GO) assay kit (Sigma-Aldrich). Glucose consumption was calculated as the difference in glucose concentration between the collected culture medium and DMEM. Absorbance was recorded at 540 nm at room temperature with a plate reader. Lactate levels were determined using a lactate assay kit (Eton Bioscience, San Diego, CA). Absorbance at 570 nm was recorded at room temperature with a plate reader. All results were normalized to the final cell number.

### Measurement of PFK activity

PFK activity was assessed using a PFK activity colorimetric assay kit (BioVision, Milpitas, CA). The reaction was performed using cell lysate (3 µg) in 100 µL of reaction buffer, which was prepared according to the kit instructions. Absorbance at 450 nm was recorded at 37 °C with a plate reader.

### Cell proliferation assay

Cells were plated at a density of 1 × 10^3^ cells per 96-well plate. Cells were treated with VEGF (20 ng/mL) for the indicated time period (days) in DMEM with 0.1% serum. Cell proliferation was measured with a Quanti-Max^TM^ WST-8 cell viability assay kit (BIOMAX, Republic of Korea). The reaction was performed by adding Quanti-Max^TM^ (10 µL per well) to culture media and then incubating for 0.5–2 h at 37 °C. Absorbance at 450 nm was recorded at 37 °C with a plate reader.

### Intracranial implantation of GBM cells in mice and histologic evaluation

We injected LN229/EGFRvIII GBM cells with or without modulation of PFKP expression or an active AKT1 mutant, intracranially into 4-week-old male athymic nude mice (five mice/group), as described previously [19]. Mice were euthanized at 5, 15, 18, or 20 days after the GBM cells were injected. The brain of each mouse was harvested, fixed in 4% formaldehyde, and embedded in paraffin. After that, histological sections (5 μm in thickness) were prepared. These sections were stained with Mayer’s hematoxylin and subsequently with eosin (H&E) (Biogenex Laboratories, San Ramon, CA). Afterward, the slides were mounted with Universal Mount (Research Genetics Huntsville, AL). Tumor formation and phenotype were determined by histological analysis of H&E-stained sections. Tumor volume was calculated with the formula of 0.5 × L × W2 (L, length; W, width). All of the mice were housed the in the Animal Central Laboratory of West China Second Hospital (Chengdu, Sichuan, China) animal facility. All experiments were performed in accordance with relevant institutional and national guidelines and regulations approved by the Institutional Animal Care and Use Committee of the State Key Laboratory of Oral Diseases, Sichuan University (Chengdu, Sichuan, China).

### IHC analysis and scoring

An IHC analysis was conducted using paraffin-embedded tissue sections. The expression of HIF-1α, β-catenin S552 phosphorylation, VEGF, and CD31 was detected with a VECTASTAIN Elite ABC kit (Vector Laboratories). Tissue sections were then incubated with 3,3′-diaminobenzidine (Vector Laboratories). Nuclei were stained with hematoxylin. Six randomly chosen fields per slide were analyzed and averaged.

Human GBM samples and clinical information were obtained from the Chinese Glioma Genome Atlas (CGGA, http://www.cgga.org.cn). This study was approved by the Ethics Committee of Capital Medical University (China). Written informed consent was obtained from each patient. The tissue sections from 25 paraffin-embedded human GBM specimens were stained with antibodies against PFKP Y64 phosphorylation, HIF-1α, β-catenin S552 phosphorylation, VEGF, or non-specific immunoglobulin as a negative control. We quantitatively scored tissue sections according to the percentage of positive cells and staining intensity as previously described [19]. We assigned the following proportion scores: 0 if 0% of the tumor cells showed positive staining, 1 if 0.1% to 1% of cells were stained, 2 if 1.1% to 10% of cells were stained, 3 if 11% to 30% of cells were stained, 4 if 31% to 70% of cells were stained, and 5 if 71% to 100% of cells were stained. We rated the intensity of staining on a scale of 0–3: 0, negative; 1, weak; 2, moderate; and 3, strong. We then combined the proportion and intensity scores to obtain a total score (range: 0–8), as described previously [19]. The use of human GBM samples and the clinical parameters were approved by the Institutional Review Board of Capital Medical University (Beijing, China).

### TCGA analyses

TCGA data were downloaded from cBioPortal (https://www.cbioportal.org). Correlation analysis between two genes was done with *Pearson*’s correlation analysis. *P*-value indicates the significance of correlation.

### Statistical analysis

All quantitative data are presented as the mean ± standard deviation (SD) of at least three independent experiments. A two-group comparison was conducted using the two-sided, two-sample Student’s *t*-test. Simultaneous comparison of more than two groups was conducted using a one-way analysis of variance (ANOVA) followed by Tukey’s post hoc tests. The SPSS statistical package version 12 (SPSS Inc., Chicago, IL) was used for all statistical analyses. Statistically significant differences were considered when *P*-values were less than 0.05.

## Results

### PFKP depletion in GBM cells results in impaired EGFR activation-induced VEGF expression in vitro and angiogenesis in vivo

**Fig. 1:**
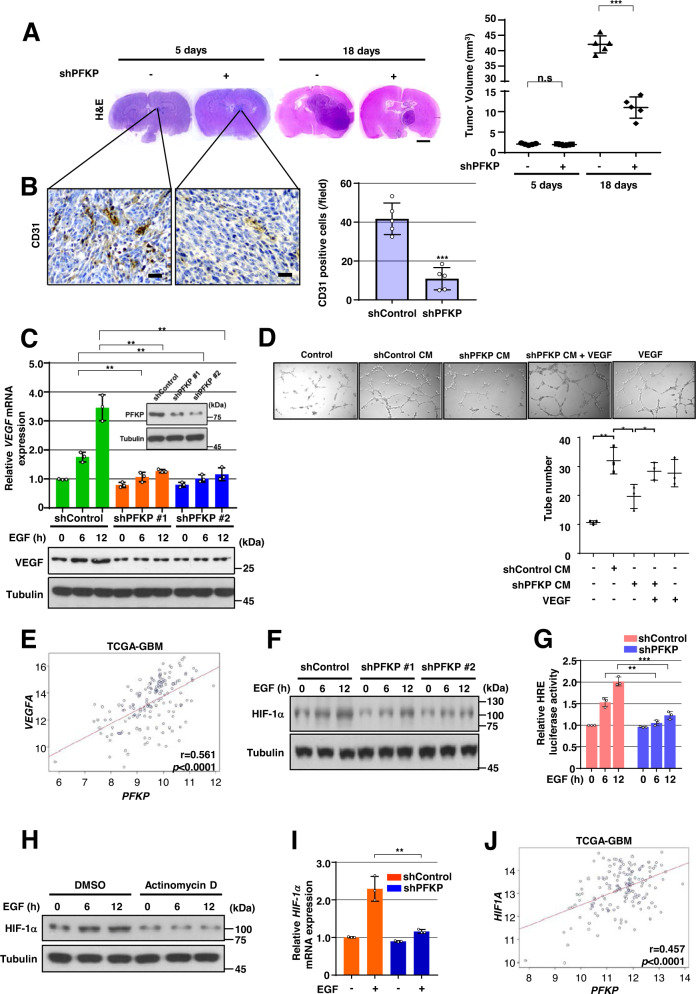
PFKP expression is required for EGFR activation-induced VEGF expression in vitro and GBM angiogenesis in vivo. WB and qRT-PCR were performed with indicated primers and antibodies, respectively (**C**, **F**, **H**, **I**). **A**, **B** Representative H&E staining images of intracranial xenografts bearing LN229/EGFRvIII cells stably expressing with or without PFKP shRNA (**A**; left panel) and quantification of tumor volumes (**A**; right panel). IHC analyses of the tumor tissues with an anti-CD31 antibody (**B**; left panel). Quantification of CD31 (**B**; right panel). Scale bar, 2 mm (**A**) and 100 μm (**B**). **C** GSCs with different shRNAs against PFKP (inside panel). Serum-starved GSCs with or without PFKP depletion were treated with EGF (100 ng/mL). **D** HUVECs were treated with or without a conditioned medium (CM) from control shRNA-expressing or PFKP shRNA-expressing LN229/EGFRvIII cells in the presence or absence of VEGF (20 ng/mL). HUVECs tube formation was observed. Representative images were acquired under an optical microscope (50×), and the tube number (/field) was quantified. **E** TCGA analysis of *PFKP* and *VEGF* mRNA expression from TCGA-GBM data (*n* = 154). **F** Serum-starved GSCs with or without PFKP depletion were treated with EGF (100 ng/mL). **G** HRE luciferase activity in GSCs with stable expression of control shRNA or PFKP shRNA was measured. **H** Serum-starved GSCs were pretreated with DMSO or actinomycin D (1 μg/mL) for 1 h and then stimulated with or without EGF (100 ng/mL). **I** Serum-starved GSCs stably expressing control shRNA, or PFKP shRNA were treated with or without EGF (100 ng/mL) for 12 h. **J** TCGA analysis of *PFKP* and *HIF-1α* mRNA expression from TCGA-GBM data (*n* = 154). Data are presented as mean ± standard deviation of three independent experiments (**A**, **C**, **D**, **G**, **I**). ***P < 0.001, based on the Student’s *t*-test.

To determine whether PFKP expression is required for the angiogenesis-mediated continued growth of GBM tumors, we depleted PFKP expression using PFKP short hairpin RNA (shRNA) in LN229 human GBM cells overexpressing constitutively active EGFRvIII mutant (LN229/EGFRvIII) (Supplementary Fig. S1A), which lacks 267 amino acids from its extracellular domain and is frequently found in GBM [13], and intracranially injected the cells into mice. After implantation and growth, tumors were excised for histologic staining. Depletion of PFKP successfully inhibited the growth of brain tumors derived from intracranially injected LN229/EGFRvIII cells at day 18 (Fig. 1A) and prolonged the survival time of the mice (Supplementary Fig. S1B). Interestingly, a reduction in PFKP expression resulted in decreased blood vessel formation as evidenced by the intensity of CD31 expression (Fig. 1B), in which the tumor sizes at an early stage (day 5) were comparable between groups injected with control shRNA-expressing and PFKP shRNA-expressing LN229/EGFRvIII cells (Fig. 1A). Consistence with these results, depletion of PFKP expression inhibited the growth of brain tumors in the group intracranially injected EGFR-expressing naïve LN229 cells (Supplementary Fig. S1C). Depletion of PFKP expression also decreased blood vessel formation. However, tumor sizes were comparable between groups injected with control shRNA-expressing and PFKP shRNA-expressing LN229 cells at an early stage (Supplementary Fig. S1C). These results suggest that PFKP plays an important role in EGFR activation-induced GBM angiogenesis in vivo.

We next determined the effect of PFKP on EGFR activation-induced VEGF expression in vitro. A reduction in PFKP expression largely reduced EGFR activation-induced *VEGF* mRNA expression and its protein expression in glioma stem cells (GSCs) (Fig. 1C), LN229 (Supplementary Fig. S1D), and U251 cells (Supplementary Fig. S1E). To further determine whether PFKP could induce angiogenesis in vitro through upregulation of VEGF expression, we performed HUVECs tube formation assay using collected conditioned medium (CM) from control shRNA-expressing or PFKP shRNA-expressing LN229/EGFRvIII cells. As shown in Fig. 1D, the suppression of PFKP significantly inhibited HUVECs tube formation of HUVECs, which was rescued by adding VEGF, showing that PFKP expression plays a role in angiogenesis by regulating VEGF expression in GBM cells. In addition, to determine the clinical relevance of PFKP-regulated VEGF expression, we analyzed The Cancer Genome Atlas (TCGA) data and revealed that the expression levels of *PFKP* were positively correlated with *VEGF* mRNA expression levels in GBMs (Fig. 1E). Our in vitro and in vivo results suggest that PFKP plays a critical role in EGFR activation-induced VEGF expression in GBM cells, thereby promoting GBM angiogenesis.

### PFKP expression is required for EGFR activation-induced HIF-1α expression and transactivation

Accumulated evidence has shown that EGFR activation increases the expression of HIF-1α in cancer cells under normoxic conditions [10, 24, 25]. To examine the effect of PFKP on EGFR activation-induced HIF-1α expression, we treated human GBM cells, including GSCs, LN229, and U251 cells, with or without expression of PFKP shRNA with EGF. Immunoblotting analysis showed that EGFR activation-induced HIF-1α expression was largely inhibited by depletion of PFKP in GSCs (Fig. 1F), LN229, and U251 cells (Supplementary Fig. S1F). In line with these findings, HRE luciferase reporter analysis showed that depletion of PFKP expression largely inhibited EGFR activation-induced HIF-1α transactivation in GSCs (Fig. 1G) and LN229/EGFRvIII cells (Supplementary Fig. S1G). Pretreatment of cancer cells with actinomycin D, a transcription inhibitor, almost abolished EGF-enhanced HIF-1α expression in GSCs (Fig. 1H), LN229, and U251 cells (Supplementary Fig. S1H), suggesting that transcriptional regulation of HIF-1α is essentially involved in response to EGFR activation. Quantitative PCR analyses showed that EGF treatment increased mRNA levels of the *HIF-1α* gene, however, such increase was attenuated by PFKP depletion in GSCs (Fig. 1I), LN229, and U251 cells (Supplementary Fig. S1I). Analyses of TCGA data showed that the expression levels of *PFKP* were positively correlated with *HIF-1α* mRNA expression levels in GBMs (Fig. 1J). Taken together, these results indicate that PFKP expression is required for EGFR activation-induced *HIF-1α* transcriptional expression and its activity in GBM cells.

### PFKP Y64 phosphorylation induces EGFR activation-enhanced *HIF-1α* transcriptional expression through SP1 transactivation

**Fig. 2:**
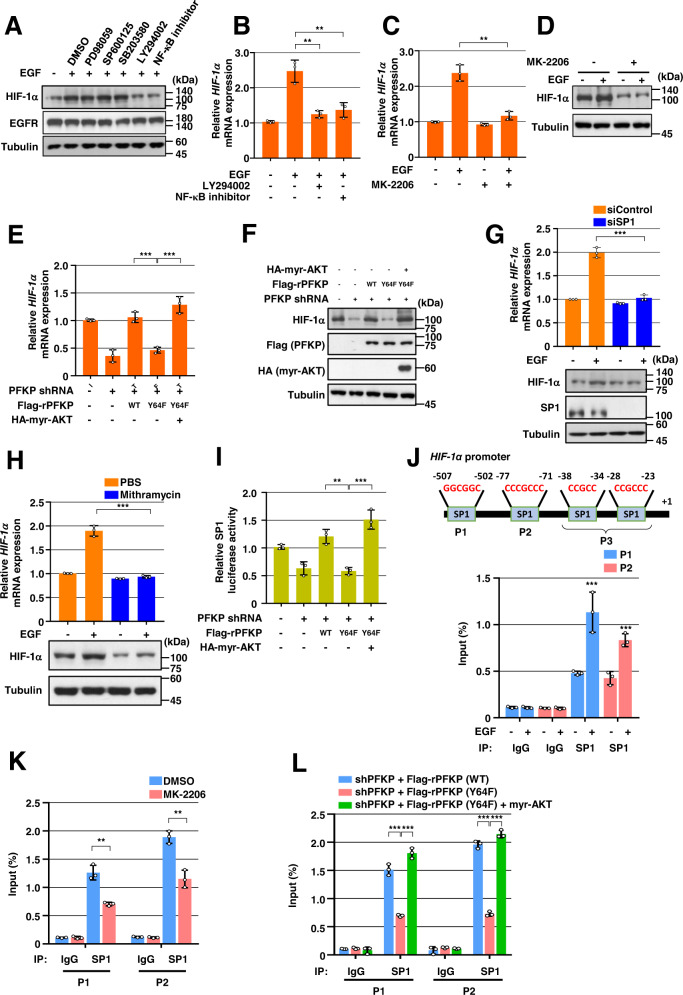
PFKP Y64 phosphorylation induces EGFR activation-enhanced *HIF-1α* transcriptional expression through SP1 transactivation. WB and qRT-PCR were performed with indicated primers and antibodies, respectively (**A**–**H**). **A** Serum-starved GSCs were pretreated DMSO, PD98059 (20 μM), SP600125 (25 μM), SB203580 (10 μM), LY294002 (20 μM), or NF-κB inhibitor (1 μM) for 1 h and then stimulated with or without EGF (100 ng/mL) for 12 h. **B**–**D** Serum-starved GSCs were pretreated DMSO, LY294002, NF-κB inhibitor (**B**), or MK-2206 (5 μM) (**C**, **D**) for 1 h and then stimulated with or without EGF (100 ng/mL) for 12 h. **E**, **F** LN229/EGFRvIII cells with or without PFKP depletion and with or without reconstituted expression of WT Flag-rPFKP or Flag-rPFKP Y64F mutant in the presence or absence of HA-myr-AKT expression. **G** GSCs were transfected with control siRNA or SP1 siRNA. **H** GSCs were treated with PBS or mithramycin (500 nM) for 12 h. **I** SP1 luciferase activity in LN229/EGFRvIII cells with or without PFKP depletion and with or without reconstituted expression of WT Flag-rPFKP or Flag-PFKP Y64F mutant in the presence or absence of HA-myr-AKT expression was measured. **J**–**L** ChIP assays were performed with anti-SP1 antibody, and real-time PCR analyses were performed with primers against the *HIF-1α* promoter. (**J**) A schematic of the putative SP1 binding site (Marked as P1–P3) on the *HIF-1α* promoter region (**J**; upper panel). GSCs were treated with or without EGF (100 ng/mL) for 12 h (**J**; bottom panel). **K** LN229/EGFRvIII cells were pretreated with DMOS or MK-2206 (5 μM) for 1 h and then treated with EGF (100 ng/ml) for 12 h. **L** LN229/EGFRvIII cells with PFKP depletion and with or without reconstituted expression of WT Flag-rPFKP or Flag-rPFKP Y64F mutant were transfected with or without HA-myr-AKT. Data are presented as mean ± standard deviation of three independent experiments (**B**, **C**, **E**, **G**, **H**–**L**). ***P < 0.001, based on the Student’s *t*-test or one-way ANOVA with Tukey’s post hoc test.

To determine how *HIF-1α* transcriptional expression is regulated by EGFR activation, we pretreated GSCs with inhibitors of early signal pathways, including ERK, JNK, p38, PI3K, or NF-κB, which successfully blocked EGF-induced ERK, c-Jun, p38, AKT, or IκBα phosphorylation, respectively (Supplementary Fig. S2A). Inhibition of PI3K/AKT or NF-κB, but not ERK, p38, or JNK, largely abrogated EGF-induced HIF-1α protein expression in GSCs (Fig. 2A). In addition, pretreatment with PI3K inhibitor or NF-κB inhibitor blocked EGF-induced *HIF-1α* mRNA expression in GSCs (Fig. 2B). In line with this result, pretreatment of GSCs, LN229, and U251 cells with MK-2206, a selective AKT1/2/3 inhibitor, blocked EGF-stimulated *HIF-1α* mRNA (Fig. 2C and Supplementary Fig. S2B) and its protein expression (Fig. 2D and Supplementary Fig. S2B). These results indicate that PI3K/AKT and NF-κB pathways are primarily involved in the regulation of the EGFR activation-induced *HIF-1α* transcriptional expression in GBM cells. In a previous report, we found that EGFR-phosphorylated PFKP Y64 promotes PI3K/AKT activation [19]. In the present study, depletion of PFKP did not alter the other EGFR activation-induced early signals, such as phosphorylation of ERK, p38, IκBα, and c-Jun in GSCs (Supplementary Fig. S2C). Thus, we excluded the NF-κB signal for the role of PFKP in EGFR activation-induced *HIF-1α* transcriptional expression. We next investigated the effect of PFKP Y64 phosphorylation on *HIF-1α* mRNA expression in response to EGFR activation. As expected, depletion of endogenous PFKP resulted in decreased *HIF-1α* mRNA (Fig. 2E) and protein (Fig. 2F) expression levels in LN229/EGFRvIII cells; such decreases were rescued by reconstituted expression of RNAi-resistant (r) WT Flag-rPFKP, but not by that of rPFKP Y64F mutant, (Fig. 2E, F). Of note, the inhibitory effect of rPFKP Y64F expression on *HIF-1α* mRNA (Fig. 2E) and protein expression (Fig. 2F) in LN229/EGFRvIII cells was restored by expression of the constitutively active AKT1 mutant (HA-myr-AKT1). These results indicate that PFKP Y64 phosphorylation enhances EGFR activation-induced *HIF-1α* mRNA and its protein expression in an AKT activation-dependent manner.

It has been reported that the transcription factor-specific protein 1 (SP1) has putative binding sites on the *HIF-1α* gene promoter [26]. However, its role and regulatory mechanism in EGFR activation-induced *HIF-1α* transcription are unknown. Depletion of SP1 (Fig. 2G and Supplementary Fig. S2D) or treatment with the SP1 inhibitor mithramycin (Fig. 2H and Supplementary Fig. S2E) decreased EGFR activation-induced *HIF-1α* mRNA and protein expression levels in GSCs and LN229/EGFRvIII cells. These data indicate that SP1 is required for *HIF-1α* transcriptional expression in response to EGFR activation. PI3K/AKT pathway is associated with increased phosphorylation of SP1 and its transcriptional activity [11, 27–30]. As PFKP Y64 phosphorylation regulates PI3K/AKT in EGFR-activated cancer cells, we investigated whether PFKP Y64 phosphorylation could modulate SP1 activity. Luciferase reporter analysis using a plasmid containing the luciferase reporter gene driven by an SP1-responsive promoter showed that the transcriptional activity of SP1 in the LN229/EGFRvIII cells expressing rPFKP Y64F in PFKP-depleted LN229/EGFRvIII cells was reduced compared with that in the cells expressing its WT counterpart, however, this reduction was abrogated by expressing an active AKT1 mutant (Fig. 2I). Next, we performed a chromatin immune precipitation (ChIP) assay with an anti-SP1 antibody to identify the exact binding site of SP1 in the promoter region of the *HIF-1α* gene in response to EGFR activation. Based on the previous report [26], we designed three sets of primers that could specifically amplify the indicated regions (P1 for −507 bp to −502 bp; P2 for −77 bp to −71 bp; and P3 for −38 bp to −23 bp, +1 indicates the first bp of exon 1) of the *HIF-1α* gene promoter (Fig. 2J; top panel). As shown in Fig. 2J (bottom panel), only the P1 and P2 regions were amplified, indicating that SP1 could specifically bind to the P1 and P2 regions of the *HIF-1α* gene promoter in response to EGFR activation in GSCs. MK-2206 treatment reduced the binding of SP1 to the promoter region of *HIF-1α* in LN229/EGFRvIII cells (Fig. 2K). Furthermore, the binding of SP1 to the promoter region of *HIF-1α* in the LN229/EGFRvIII cells expressing rPFKP Y64F in PFKP-depleted LN229/EGFRvIII cells was reduced compared with that in the cells expressing its WT counterpart, however, this reduction was alleviated by expressing an active AKT1 mutant (Fig. 2L). These results strongly suggest that the PFKP Y64 phosphorylation can regulate EGFR activation-enhanced *HIF-1α* transcriptional expression through the AKT/SP1 pathway in GBM cells.

### PFKP Y64 phosphorylation induces VEGF expression through HIF-1 α expression and β-catenin Ser552 phosphorylation in response to EGFR activation

**Fig. 3:**
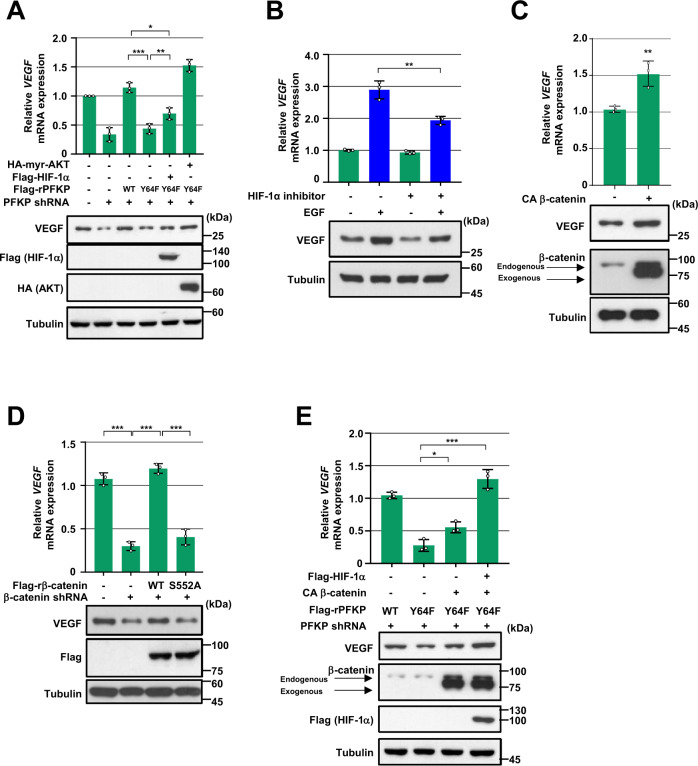
PFKP Y64 phosphorylation induces VEGF expression through HIF-1 α expression and β-catenin Ser552 phosphorylation in response to EGFR activation. WB and qRT-PCR were performed with indicated primers and antibodies, respectively (**A**–**E**). **A** LN229/EGFRvIII cells with or without PFKP depletion and with or without reconstituted expression of WT Flag-rPFKP or Flag-rPFKP Y64F mutant were transfected with or without Flag-HIF-1α and HA-myr-AKT. **B** Serum starved LN229 cells were pretreated with DMSO or HIF inhibitor (10 μM) for 1 h and then treated with EGF (100 ng/mL) for 12 h. **C** LN229 cells were transfected with a control vector or CA β-catenin. **D** LN229/EGFRvIII cells with stable expression of β-catenin shRNA or a control shRNA were reconstituted with or without WT rβ-catenin or rβ-catenin S552A mutant. **E** PFKP-depleted LN229/EGFRvIII cells with or without reconstituted expression of WT Flag-rPFKP or Flag-rPFKP Y64F mutant were transfected with or without CA β-catenin and Flag- HIF-1α. Data are presented as mean ± standard deviation of three independent experiments (**A**–**E**). **P < 0.01; ***P < 0.001, based on the Student’s *t*-test or one-way ANOVA with Tukey’s post hoc test.

We next determined the role of PFKP Y64 phosphorylation in EGFR activation-induced VEGF expression. Depletion of PFKP resulted in decreased EGFR activation-induced *VEGF* mRNA and protein expression levels in LN229/EGFRvIII cells (Fig. 3A). Such decreases were rescued by reconstituted expression of WT Flag-rPFKP, but not by the expression of the rPFKP Y64F mutant (Fig. 3A), suggesting a crucial role of PFKP Y64 phosphorylation in the EGFR activation-induced VEGF expression. Interestingly, the inhibitory effect of rPFKP Y64F on VEGF expression in LN229/EGFRvIII cells was not fully rescued by exogenous expression of HIF-1α (Fig. 3A). In addition, EGFR activation still induced VEGF expression in LN229 (Fig. 3B) and U251 cells (Supplementary Fig. S3A) even when we blocked HIF-1α transactivation by treatment with a HIF-1α inhibitor (Supplementary Fig. S3B). These results suggest that PFKP Y64 phosphorylation can upregulate VEGF expression in HIF-1α-dependent and -independent mechanisms in response to EGFR activation.

It has been reported that β-catenin regulates VEGF expression in colon cancer [31]. Consistent with this report, expression of the constitutively active β-catenin (CA β-catenin) mutant increased *VEGF* mRNA expression and its protein expression levels in LN229 cells (Fig. 3C). In contrast, depletion of β-catenin reduced *VEGF* mRNA and protein expression levels in LN229/EGFRvIII cells (Fig. 3D). It has been shown that AKT can directly phosphorylate β-catenin at S552 to promote the nuclear translocation and activation of β-catenin [32]. Reconstituted expression of WT rβ-catenin, but not the rβ-catenin S552A mutant, successfully restored the reduction of VEGF expression levels in the β-catenin-depleted LN229/EGFRvIII cells (Fig. 3D), suggesting that AKT-dependent β-catenin activation is instrumental for EGFR activation-increased VEGF expression. We have previously reported that PFKP can induce AKT-mediated β-catenin S552 phosphorylation and subsequent β-catenin transactivation in a PFKP Y64 phosphorylation-dependent manner [33]. Because PFKP Y64F-reduced β-catenin S552 phosphorylation and subsequent β-catenin transactivation were involved in the reduced VEGF expression in PFKP-depleted LN229/EGFRvIII cells (Fig. 3E; second lane), we ectopically introduced expression of CA β-catenin and found that expression of CA β-catenin partially rescued the PFKP Y64F-reduced VEGF expression (Fig. 3E; third lane), which was fully rescued by additional exogenous expression of HIF-1α (Fig. 3E; fourth lane). In line with these results, the expression of an active AKT1 mutant fully rescued the inhibitory effect of PFKP Y64F on VEGF expression (Fig. 3A; sixth lane). These results demonstrate that PFKP Y64 phosphorylation plays critical roles in EGFR activation-induced VEGF expression through AKT/SP1-mediated HIF-1α expression and AKT-mediated β-catenin S552 phosphorylation.

### PFKP Y64 phosphorylation induces HIF-1 α expression, β-catenin S552 phosphorylation, and VEGF expression and promotes blood vessel formation in vivo

**Fig. 4:**
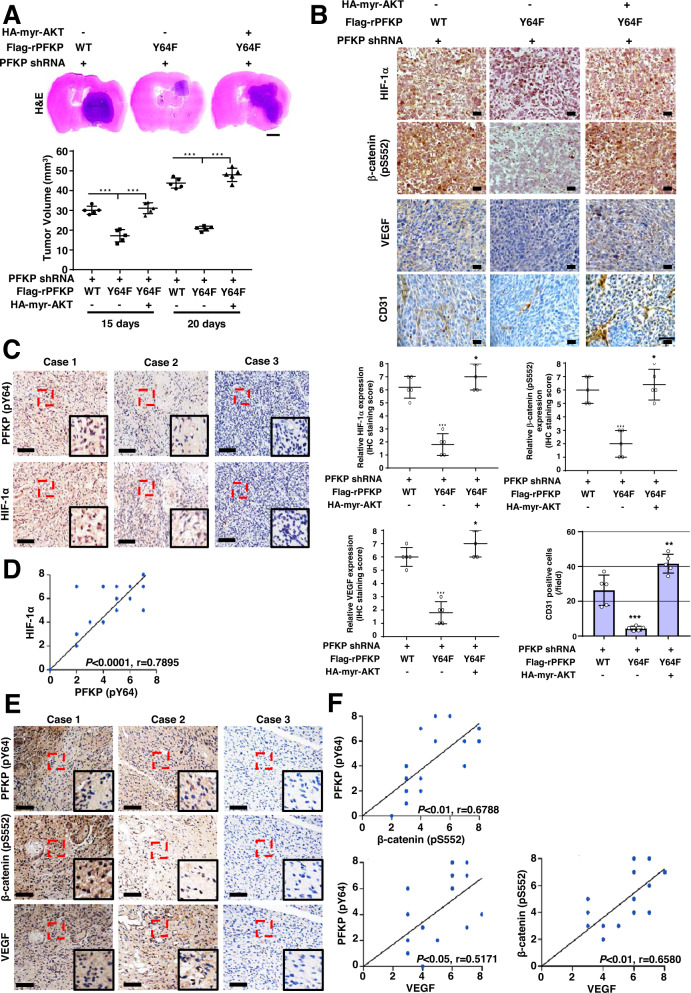
PFKP Y64 phosphorylation induces HIF-1 α expression, β-catenin S552 phosphorylation, and VEGF expression and promotes blood vessel formation in vivo. **A** Representative H&E staining images of intracranial xenografts bearing PFKP-depleted LN229/EGFRvIII cells with or without reconstituted expression of WT Flag-rPFKP or Flag-rPFKP Y64F mutant with or without myr-AKT1 (**A**; upper panel) and quantification of tumor volumes (**A**; bottom panel). Scale bar, 2 mm. **B** IHC analyses of tumor tissues with the indicated antibodies (upper panel). Quantification of indicated IHC staining (bottom panel). Scale bar, 100 μm. **C**, **E** IHC staining of human GBM specimens was performed with indicated antibodies (*n* = 25). Representative images from the staining of three different specimens are shown. Scale bar, 100 μm. **D**, **F** The IHC stains were scored, and correlation analyses were performed. The Pearson correlation test was used. Note that the scores of some samples overlapped. ***P < 0.001, based on one-way ANOVA with Tukey’s post hoc test.

We next intracranially injected PFKP-depleted LN229/EGFRvIII cells with reconstituted expression of WT rPFKP or rPFKP Y64F mutant with or without an active AKT1 mutant into athymic nude mice. Growth of brain tumors (Fig. 4A), HIF-1α expression, β-catenin S552 phosphorylation, VEGF expression, and blood vessel formation (Fig. 4B) in mice implanted with PFKP-depleted LN229/EGFRvIII cells expressing rPFKP Y64F were decreased compared with those in mice implanted with the cells expressing its WT counterpart, and this reduction was restored by expression of an active AKT1 mutant (Fig. 4A, B). In addition, the prolonged survival time of the mice bearing PFKP-depleted LN229/EGFRvIII cells expressing rPFKP Y64F was reversed by the expression of an active AKT1 mutant (Supplementary Fig. S4). These results indicate that PFKP Y64 phosphorylation-induced AKT activation plays critical roles in EGFR activation-induced HIF-1α expression, β-catenin S552 phosphorylation, VEGF expression, blood vessel formation, and brain tumor growth.

To determine the clinical significance of PFKP Y64 phosphorylation-mediated HIF-1α expression, β-catenin S552 phosphorylation, and VEGF expression, we analyzed human primary GBM specimens through immunohistochemical (IHC) staining. PFKP Y64 phosphorylation levels were positively correlated with HIF-1α expression, β-catenin S552 phosphorylation, and VEGF expression (Fig. 4C, E), and these correlations were statistically significant (Fig. 4D, F).

### VEGF induces PFKP expression, PFK enzyme activity, aerobic glycolysis, and proliferation in GBM cells

**Fig. 5:**
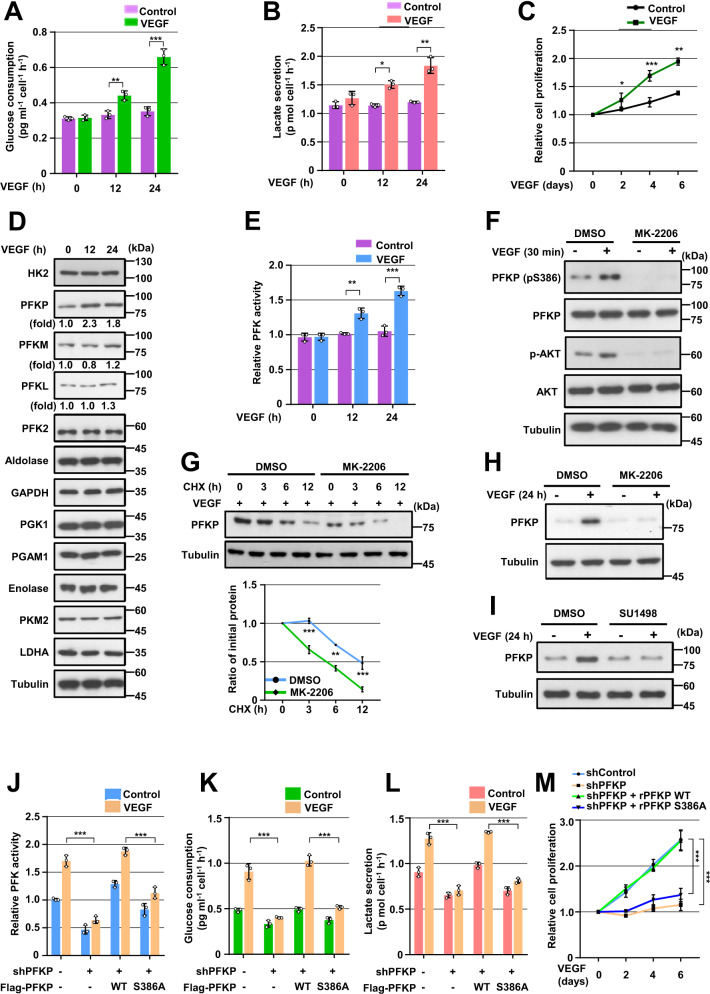
VEGF induces PFKP expression, PFK enzyme activity, aerobic glycolysis, and proliferation in GBM cells. WB and qRT-PCR were performed with indicated primers and antibodies, respectively (**D**, **F**–**I**). **A**, **B** Serum-starved GSCs were treated with VEGF (20 ng/mL). Glucose consumption (**A**) and lactate secretion (**B**) were analyzed. **C** GSCs in 0.1% serum medium were treated with VEGF (20 ng/mL). WST-8 assay was then performed. **D**, **E** Serum-starved GSCs were treated with VEGF (20 ng/mL). Indicated protein expression levels (**D**) and PFK enzymatic activity (**E**) were measured. **F** Serum-starved GSCs were pretreated with DMSO or MK-2206 (5 μM) for 1 h and then stimulated with VEGF (20 ng/mL) for 30 min. **G** Serum-starved GSCs were pretreated with VEGF (20 ng/mL) for 1 h and then treated with cycloheximide (CHX;100 μg/mL) in the presence of DMSO or MK-2206 (5 μM). Quantification of PFKP levels relative to tubulin is shown (bottom panel). **H** Serum-starved GSCs were pretreated with DMSO or MK-2206 (5 μM) for 1 h and then stimulated with or without VEGF (20 ng/mL) for 24 h. **I** Serum-starved GSCs were pretreated with DMSO or SU1498 (30 μM) for 1 h and then stimulated with or without VEGF (20 ng/mL) for 24 h. **J**–**L** LN229 cells with or without expression of PFKP shRNA and with or without the reconstituted expression of WT Flag-rPFKP or Flag-rPFKP S386A were cultured in serum-free DMEM with or without VEGF (20 ng/mL) for 24 h. PFK enzymatic activity (**J**), glucose consumption (**K**), and lactate secretion (**L**) were then analyzed. **M** LN229 cells with or without the expression of PFKP shRNA and with or without the reconstituted expression of WT Flag-rPFKP or Flag-rPFKP S386A were cultured in 0.1% serum medium with or without VEGF (20 ng/mL). WST-8 assay was then performed. Data are presented as mean ± SD of three independent experiments (**A**–**C, E, G, J**–**M**). *P < 0.05; **P < 0.01; ***P < 0.001, based on the Student’s *t*-test or one-way ANOVA with Tukey’s post hoc test.

We next examined the role and mechanism of angiogenesis-independent functions of VEGF in GBM cells. Interestingly, VEGF stimulation successfully induced glucose consumption (Fig. 5A and Supplementary Fig. S5A), lactate secretion (Fig. 5B and Supplementary Fig. S5B), and proliferation (Fig. 5C and Supplementary Fig. S5C) in GSCs, LN229, and A172 cells. To investigate which enzymes were regulated in GBM cells during VEGF-enhanced aerobic glycolysis, we analyzed protein expression profiles of glycolytic enzymes using immunoblotting. Protein expression levels of glycolytic enzymes, including hexokinase 2 (HK2), PFKM, PFK2, aldolase, glyceraldehyde 3-phosphate dehydrogenase (GAPDH), phosphoglycerate kinase 1 (PGK1), phosphoglycerate mutase 1 (PGAM1), enolase, pyruvate kinase M2 (PKM2), and lactate dehydrogenase A (LDHA) in GSCs, LN229, and A172 cells was not altered by VEGF stimulation (Fig. 5D and Supplementary Fig. S5D). However, VEGF strongly upregulated PFKP expression in several GBM cells, including GSCs, LN229, A172, LN18, U373MG, and T98G cells (Fig. 5D, Supplementary Fig. S5D, and S5E). It marginally induced PFKL expression (Fig. 5D and Supplementary Fig. S5D). Increased expression of PFKP plays a role in the regulation of PFK activity [18]. As expected, VEGF stimulation enhanced total PFK enzyme activities in GSCs, LN229, and A172 cells (Fig. 5E and Supplementary Fig. S5F).

We have previously reported that AKT phosphorylates PFKP at S386 to inhibit TRIM21-mediated degradation of PFKP, resulting in increased PFKP expression [18]. VEGF successfully induced AKT phosphorylation and PFKP S386 phosphorylation in GSCs, LN229, and A172 cells (Fig. 5F and Supplementary Fig. S5G), which was inhibited by pretreatment with an AKT inhibitor MK-2206. Furthermore, the VEGF-induced half-lives of endogenous PFKP (Fig. 5G and Supplementary Fig. S5H) and PFKP expression (Fig. 5H and Supplementary Fig. S5I) in GSCs, LN229, and A172 cells were largely decreased by pretreatment with MK-2206. The VEGF-induced PFKP expression was blocked by pretreatment of SU1498, a VEGFR2 tyrosine kinase inhibitor, in GBM cells (Fig. 5I and Supplementary Fig. S5J), indicating VEGFR-dependent VEGF signaling. To investigate the role of PFKP S386 phosphorylation in VEGF-induced PFK activity, aerobic glycolysis, and proliferation, we depleted endogenous PFKP in LN229 and A172 cells and reconstituted the expression of WT Flag-rPFKP or Flag-rPFKP S386A in these cells. The depletion of PFKP largely impaired VEGF-induced PFK activity (Fig. 5J and Supplementary Fig. S5K), glucose consumption (Fig. 5K and Supplementary Fig. S5L), lactate production (Fig. 5L and Supplementary Fig. S5M), and proliferation (Fig. 5M and Supplementary Fig. S5N), and this inhibition was rescued by the expressing WT rPFKP, but not by expressing rPFKP S386A mutant in LN229 cells (Fig. 5J-M) and A172 cells (Supplementary Fig. 5K-5N). Taken together, these results indicate that VEGF can induce PFKP expression in a PFKP S386 phosphorylation-dependent manner, leading to enhanced PFK enzyme activity, aerobic glycolysis, and proliferation in GBM cells.

## Discussion

**Fig. 6:**
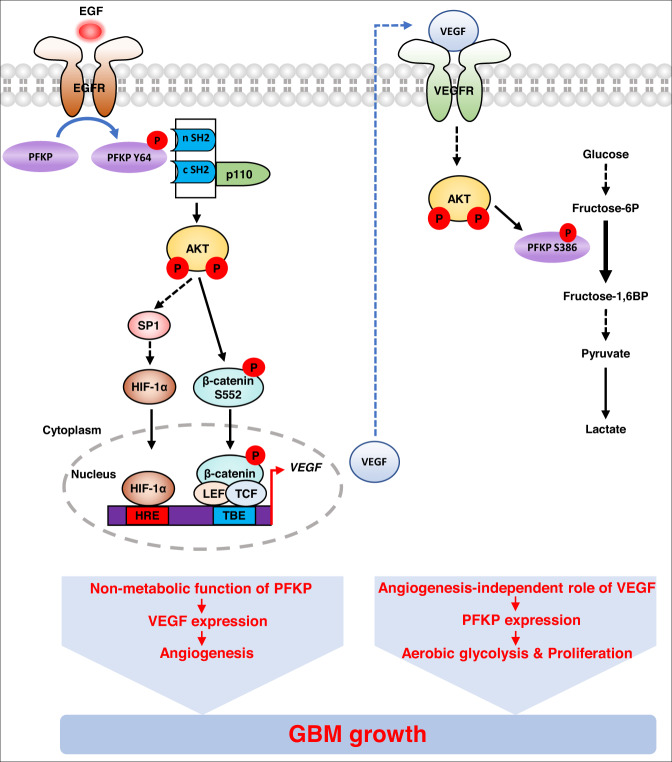
Reciprocal regulation between PFKP and VEGF promotes GBM tumor growth. A schematic of the proposed reciprocal regulation that occurs between PFKP and VEGF for regulating GBM tumor growth. HRE hypoxia response element, TBE TCF binding element.

Recent studies have shown that some metabolic enzymes and metabolites in cancer cells have been found to have noncanonical or nonmetabolic functions, which are distinct from their original roles in metabolism, and that these play critical roles in a wide spectrum of instrumental cellular activities, including metabolism, and gene expression [15]. Thus, metabolism can be connected in complex ways to multiple cellular processes in human cancer. Here, we demonstrated that EGFR-phosphorylated PFKP Y64 can induce VEGF expression directly through AKT activation-mediated β-catenin S552 phosphorylation and indirectly through AKT/SP1-mediated *HIF-1α* transcriptional expression, thereby enhancing blood vessel formation in GBM tumors (Fig. 6). Furthermore, VEGF induced the phosphorylation of PFKP S386, resulting in increased PFKP expression, PFK enzyme activity, aerobic glycolysis, and proliferation in GBM cells (Fig. 6). Our strong evidence highlights that the nonmetabolic function of PFKP can induce angiogenesis and that angiogenesis-independent role of VEGF can induce aerobic glycolysis and cancer proliferation, which are mediated by reciprocal regulation between PFKP and VEGF to promote GBM tumor growth (Fig. 6).

Previous studies have shown that HIF-1α accumulation results from intrinsic factors, including gain-of-function of oncoproteins (such as EGFR) and/or loss-of-function of tumor suppressors (such as PTEN) [10, 25]. We and others [10, 25] have shown that increased activity of EGFR, which correlates with poor clinical prognosis in many types of tumors [14], is associated with increased HIF-1α expression in a manner distinct from that mediated by hypoxia [34]. Growth factor stimulation induces translation of HIF-1α through activation of the phosphoinositol-3-kinase (PI3K)/AKT/mammalian target of rapamycin (mTOR) pathway [35, 36]. We could not exclude the possibility that PFKP can regulate HIF-1α translation, because depletion of PFKP resulted in inhibited AKT-dependent mTOR S2448 phosphorylation (data not shown), which reflects mTOR activity required for HIF-1α translation [35, 36] in response to EGFR activation. Taken together, our data and the results of others strongly suggest that the PI3K/AKT pathway could control HIF-1α expression at both the transcriptional and translational levels under normoxic conditions. Our data defined a central role of PFKP Y64 phosphorylation in EGFR activation-induced HIF-1α upregulation in GBM cells.

Our previous in vivo tumor xenograft experiments have revealed that PFKP is required for brain tumor growth [18, 19]. In the present study, we found that PFKP abrogation decreased blood vessel formation in vivo. Thus, we evaluated a close association between PFKP and VEGF expression. In this study, we found that blockade of HIF-1α transactivation marginally inhibited EGFR activation-induced VEGF expression. In addition, exogenous HIF-1α expression could not fully rescue the inhibitory effect of PFKP Y64F mutant expression on EGFR activation-induced VEGF expression, suggesting that another pathway (i.e., a HIF-1α-independent mechanism) might also play a role in regulating PFKP Y64 phosphorylation-induced VEGF expression in response to EGFR activation. Recently, we have reported that levels of activated nuclear β-catenin are positively correlated with glioma grades [37] and that PFKP plays an instrumental role in EGFR activation-induced β-catenin transactivation in GBM cells [33]. Consistent with a previous report showing that β-catenin can directly induce VEGF expression in human colon cancer cells [31], we found that β-catenin activation by AKT-mediated β-catenin S552 phosphorylation had an important function in EGFR activation-induced *VEGF* transcription in a PFKP Y64 phosphorylation-dependent manner in GBM. Taken together, these observations demonstrate that transcriptional activities of both HIF-1α and β-catenin are required for EGFR activation-induced VEGF expression in GBM cells, in which PFKP Y64 phosphorylation plays a central role. Our in vivo data also provides strong evidence that EGFR-phosphorylated PFKP Y64 can induce expression levels of HIF-1α, β-catenin S552 phosphorylation, and VEGF, leading to enhanced vascularization of tumors in the GBM xenograft. The clinical significance of these findings was evidenced by the positive correlations of PFKP Y64 phosphorylation with HIF-1α expression, β-catenin S552 phosphorylation, and VEGF expression in human GBM specimens.

Secreted VEGF acts not only on vascular endothelial cells *via* paracrine [1] for angiogenesis-dependent tumor growth but also on tumor cells *via* autocrine for angiogenesis-independent tumor growth. Co-expression of VEGF and VEGF receptors (VEGFRs) is commonly observed in a variety of tumor cells, including GBM [38]. It enables VEGF/VEGFRs autocrine signaling within a tumor mass to regulate proliferation and tumor growth [38–43]. In this study, we provided important evidence that VEGF could cause metabolic alterations mainly through PFKP upregulation in GBM cells. We have previously reported that AKT-mediated phosphorylation of PFKP at S386 can inhibit TRIM21-mediated polyubiquitylation and degradation of PFKP [18]. Here, we found that VEGF signaling activated AKT to induce PFKP S386 phosphorylation, resulting in enhanced PFKP expression, PFK enzyme activity, and aerobic glycolysis with subsequent proliferation in GBM cells. These findings collectively suggest that the VEGF is required for cell-autonomous tumor cell proliferation in human GBM cells in a PFKP expression-dependent manner, at least in part.

Microvascular proliferation is a pathologic hallmark of GBM. GBM is one of the most neovascularized solid tumors with disorganized vessels due to the high expression of VEGF and its signaling via endothelial tyrosine kinase receptor VEGFR2 [44]. It is well reported that expression levels of VEGF and its receptor are correlated with the histologic grade of gliomas [45, 46]. Thus, an antiangiogenic approach against GBM has been evaluated in clinical trials using monoclonal antibodies and tyrosine kinase inhibitors. However, results of recent clinical trials revealed that Bevacizumab (Avastin®-Beva), a humanized anti-VEGF monoclonal antibody, and Cediranib, an orally available pan-VEGFR tyrosine kinase inhibitor, failed to show any effects on GBM patients’ overall survival [47–50]. Furthermore, Bevacizumab-treated GBM tumors acquired resistance and even more aggressive and invasive phenotypes in part by increasing aerobic glycolysis [51, 52]. Our previous and present findings showed that metabolic or non-metabolic functions of PFKP can induce aerobic glycolysis [18, 19] and that non-metabolic functions of PFKP can induce VEGF expression. Given that PFKP plays critical roles in aerobic glycolysis and angiogenesis, blockage of glycolysis and VEGF expression by targeting PFKP expression or PFKP Y64 phosphorylation could be new strategies to potentiate the therapeutic effect of antiangiogenic treatment.

In summary, our study demonstrates a reciprocal action between PFKP and VEGF signaling for the first time. Such reciprocal action between PFKP and VEGF signaling might contribute to their overexpression within a tumor mass. This finding highlights novel mechanisms underlying a nonmetabolic function of glycolytic enzyme PFKP-mediated angiogenesis and angiogenesis-independent functions of VEGF-mediated metabolic regulation, which are integrated with mutual regulation to promote GBM tumor growth. These findings underscore that cancer cells’ fundamental biological processes, metabolism, and other cellular activities are integrated and mutually regulated to promote tumor development.

## Supplementary information

Supplemetal information

AJ Checklist

## Data Availability

All data generated or analyzed during this study are included in this published article.
