# Supplementary material for: Mutual regulation between phosphofructokinase 1 platelet isoform and VEGF promotes glioblastoma tumor growth
Source: Cell Death Dis. 2022 Nov 26;13(11):1002. doi: 10.1038/s41419-022-05449-6 (PMC9701207; doi:10.1038/s41419-022-05449-6)
Supplement: Supplementary file 1 — Supplemetal information [file 41419_2022_5449_MOESM1_ESM.pdf]

Supplementary Figure 1

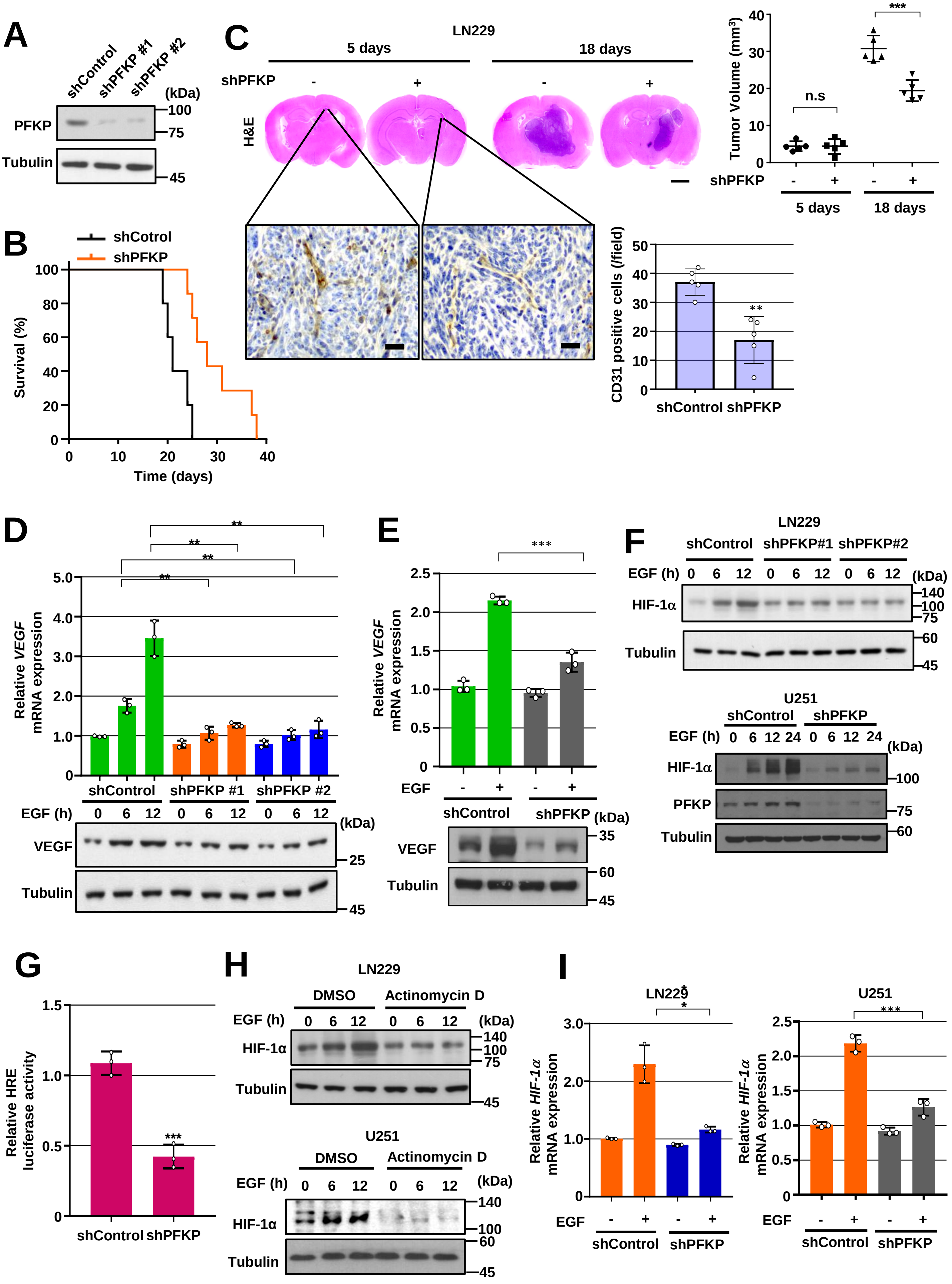

Supplementary Figure 2

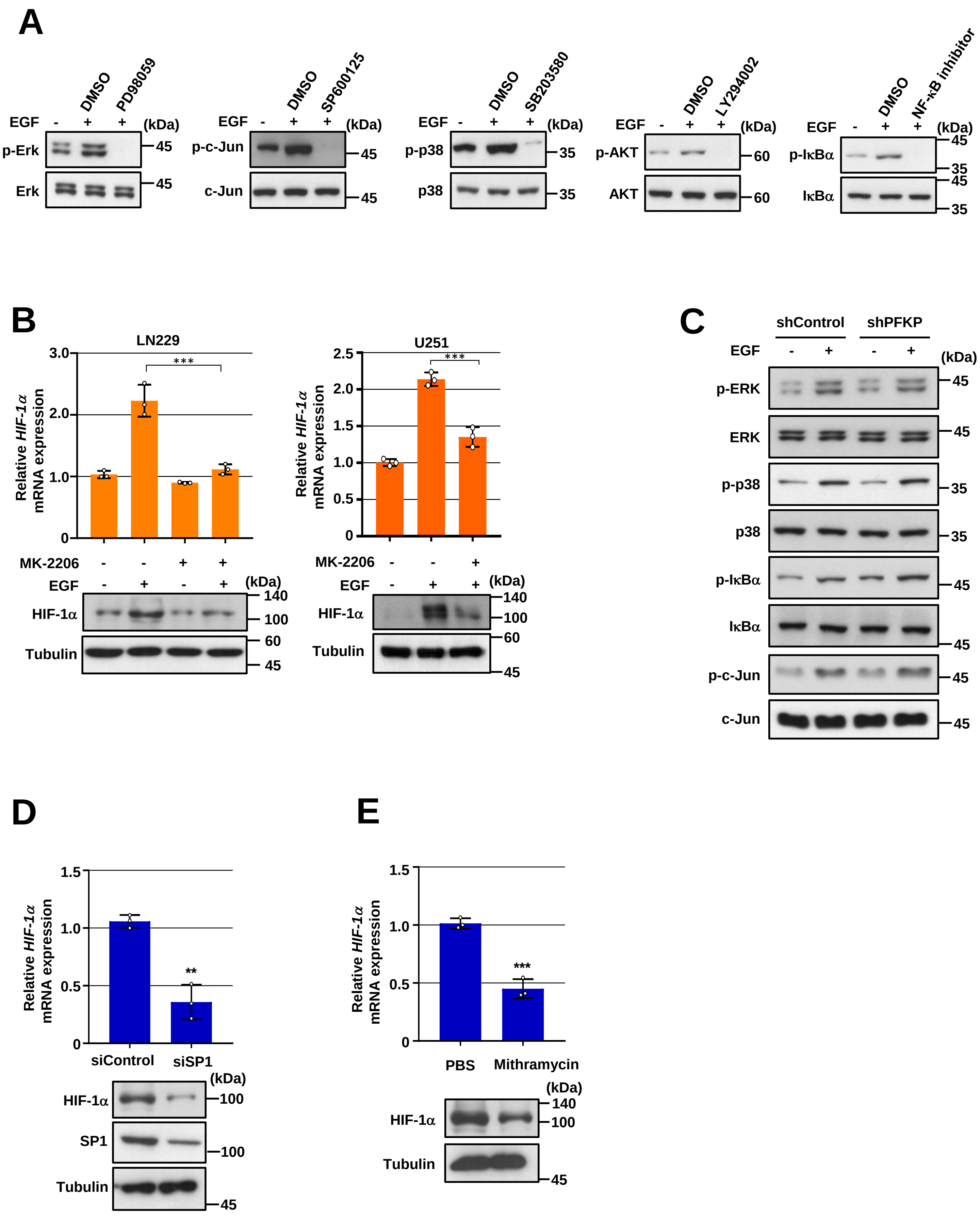

Supplementary Figure 3

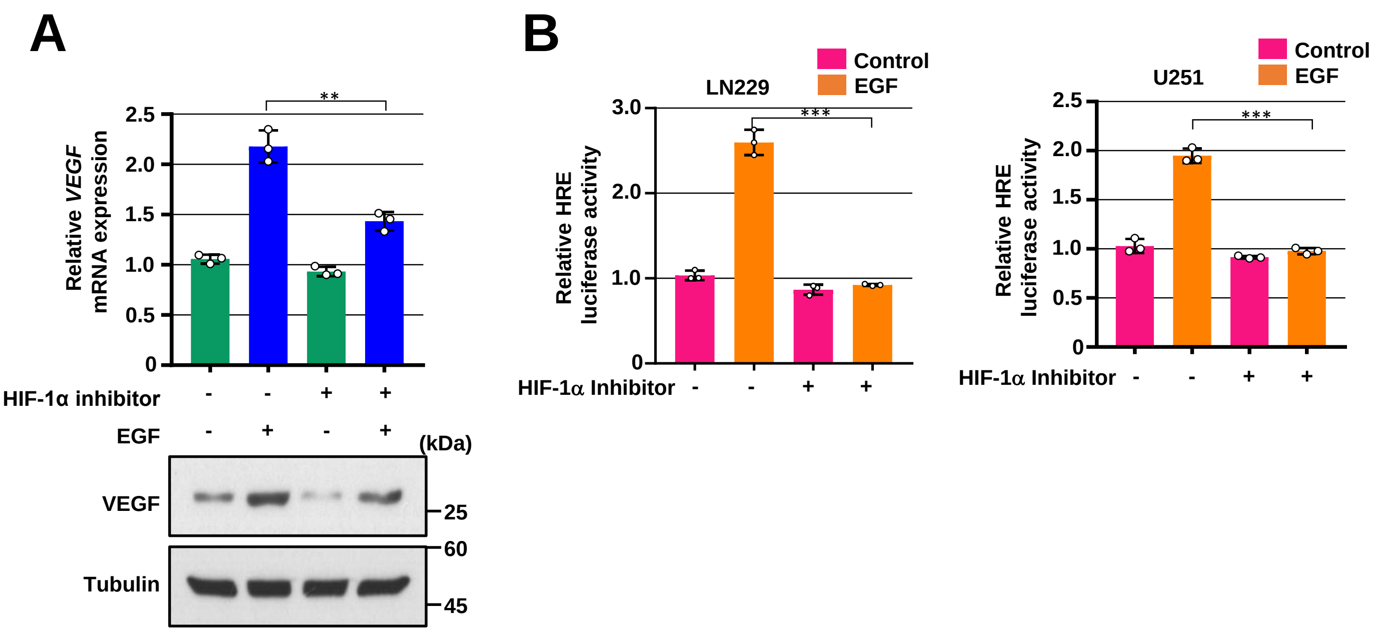

Supplementary Figure 4

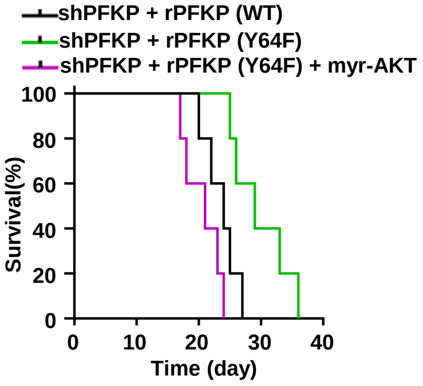

Supplementary Figure 5

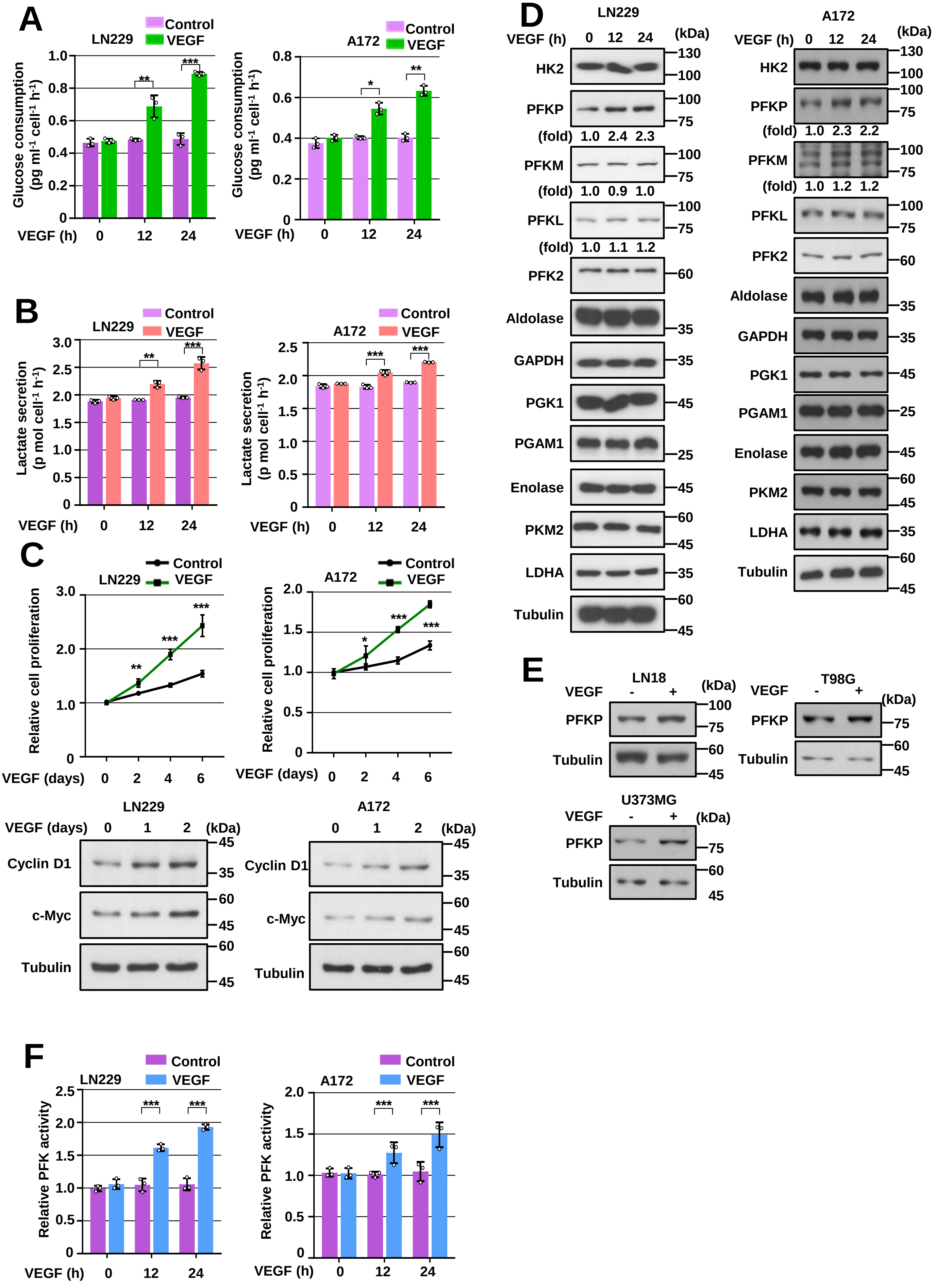

Supplementary Figure 5

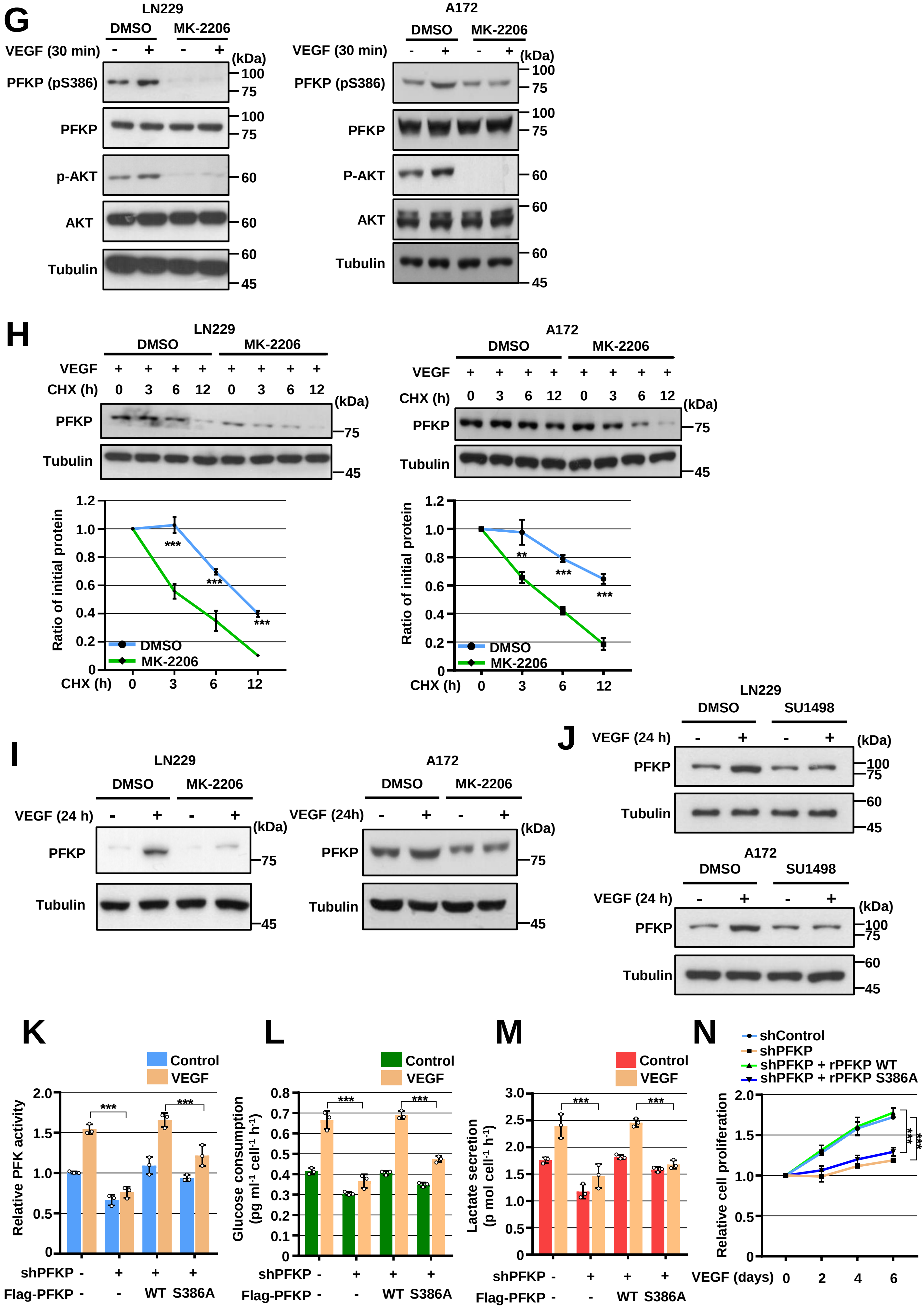

Supplementary Figure 6

Figure 1

C

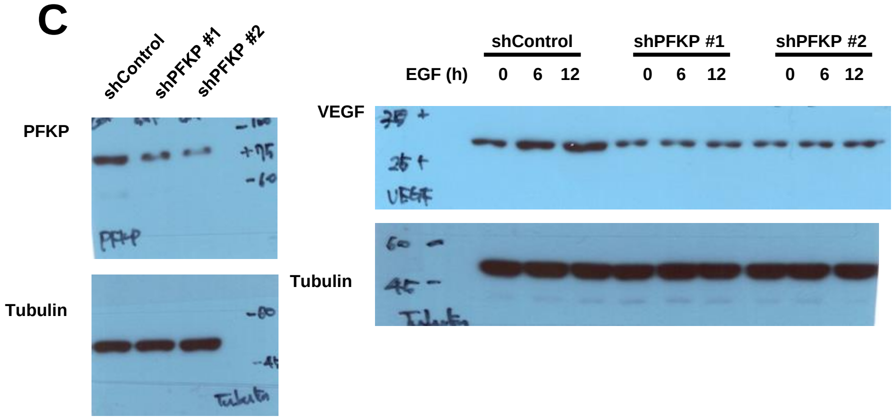

F

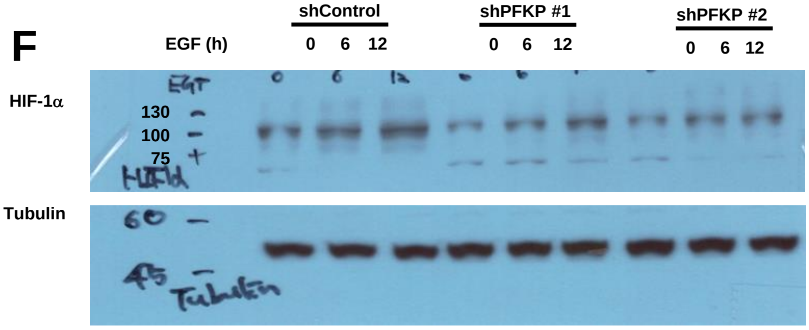

H

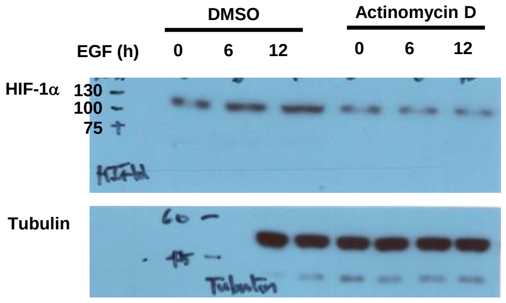

Figure 2

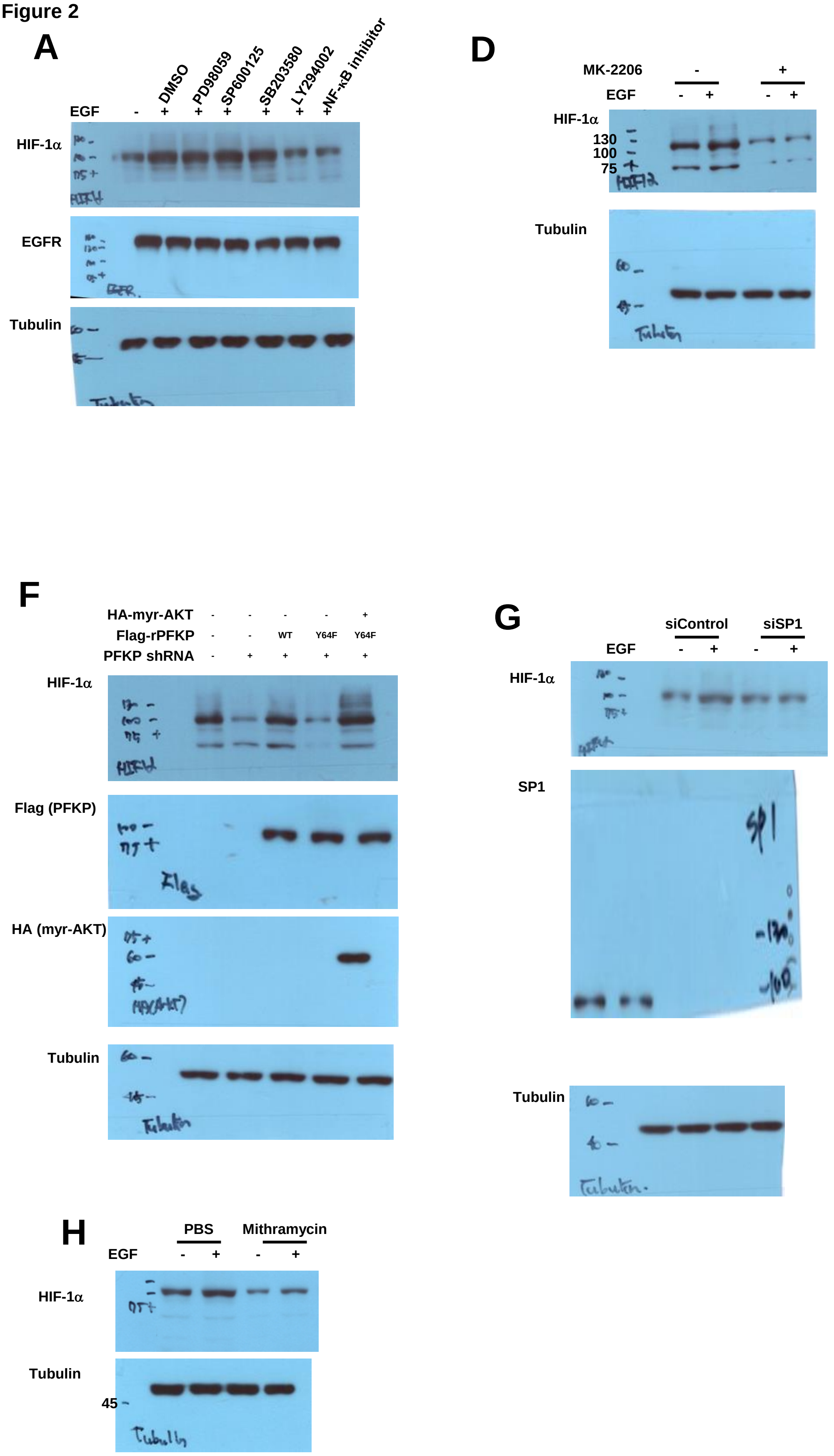

A
EGF - + + + + + +
DMSO
PD98059
SP600125
SB203580
LY294002
NF-κB inhibitor
HIF-1α
EGFR
Tubulin
D
MK-2206 - +
EGF - + - +
HIF-1α
130
100
75
Tubulin
F
HA-myr-AKT - - - - +
Flag-rPFKP - - WT Y64F Y64F
PFKP shRNA - + + + +
HIF-1α
Flag (PFKP)
HA (myr-AKT)
Tubulin
G
siControl siSP1
EGF - + - +
HIF-1α
SP1
Tubulin
H
PBS Mithramycin
EGF - + - +
HIF-1α
Tubulin
45

Figure 3

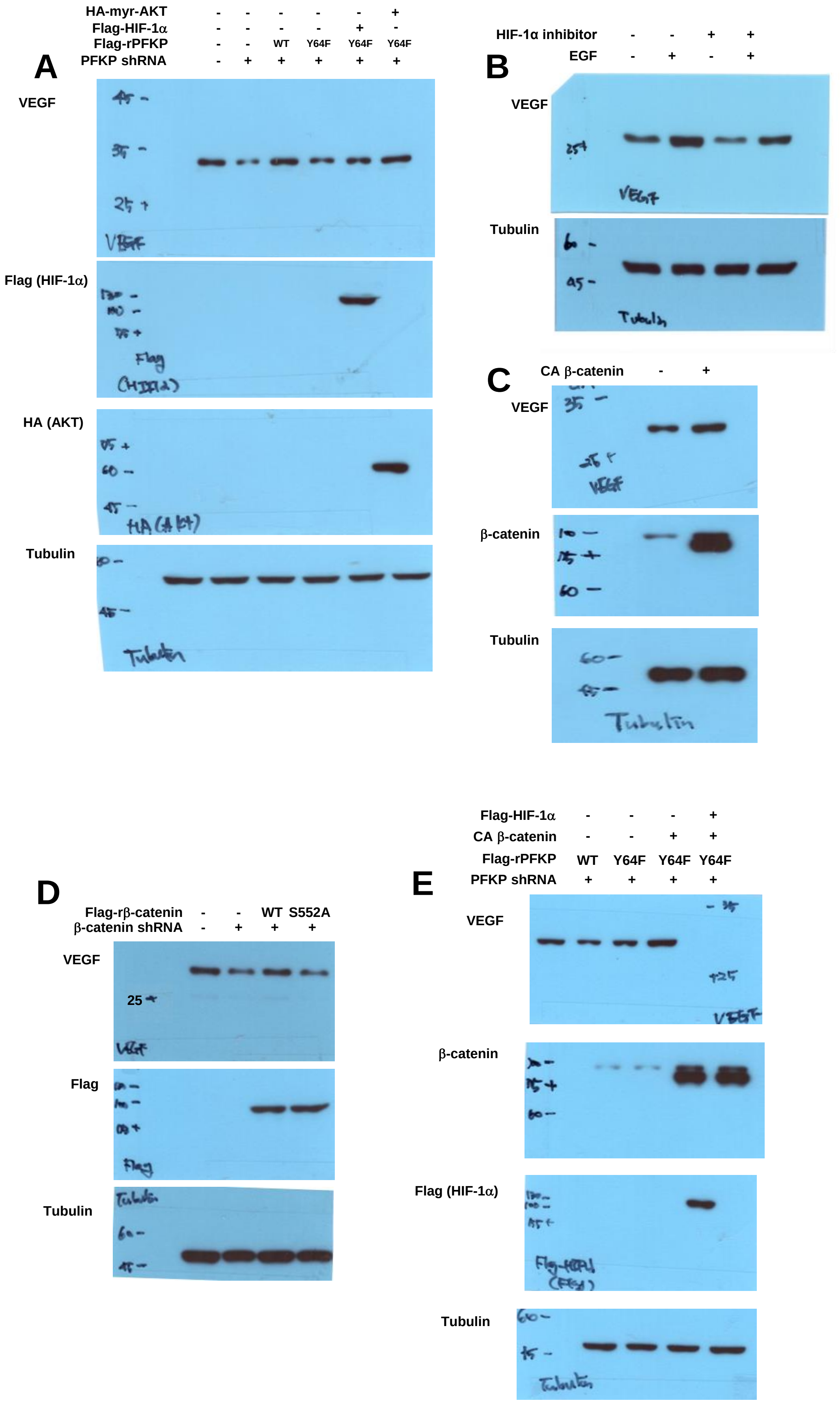
A
HA-myr-AKT - - - - - +
Flag-HIF-1α - - - - + -
Flag-rPFKP - - WT Y64F Y64F Y64F
PFKP shRNA - + + + + +
VEGF
Flag (HIF-1α)
HA (AKT)
Tubulin
B
HIF-1α inhibitor - - + +
EGF - + - +
VEGF
Tubulin
C
CA β-catenin - +
VEGF
β-catenin
Tubulin
D
Flag-rβ-catenin - - WT S552A
β-catenin shRNA - + + +
VEGF
25
Flag
Tubulin
E
Flag-HIF-1α - - - +
CA β-catenin - - + +
Flag-rPFKP WT Y64F Y64F Y64F
PFKP shRNA + + + +
VEGF
β-catenin
Flag (HIF-1α)
Tubulin

Figure 5

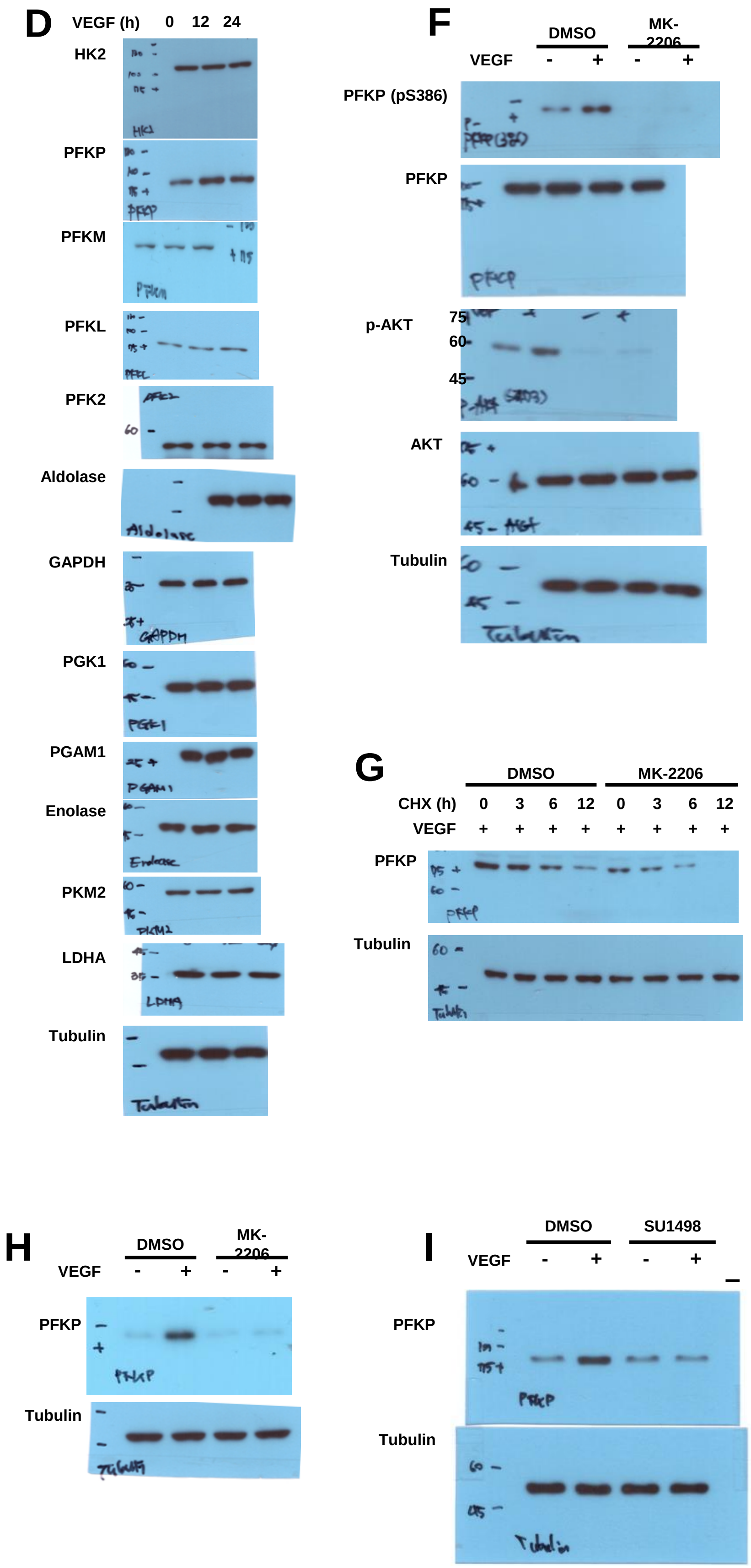
D
VEGF (h)
0
12
24
HK2
PFKP
PFKM
PFKL
PFK2
Aldolase
GAPDH
PGK1
PGAM1
Enolase
PKM2
LDHA
Tubulin
F
DMSO
MK-2206
VEGF
-
+
-
+
PFKP (pS386)
PFKP
p-AKT
75
60
45
AKT
Tubulin
G
DMSO
MK-2206
CHX (h)
0
3
6
12
0
3
6
12
VEGF
+
+
+
+
+
+
+
+
PFKP
Tubulin
H
DMSO
MK-2206
VEGF
-
+
-
+
PFKP
Tubulin
I
DMSO
SU1498
VEGF
-
+
-
+
PFKP
Tubulin

Figure S1

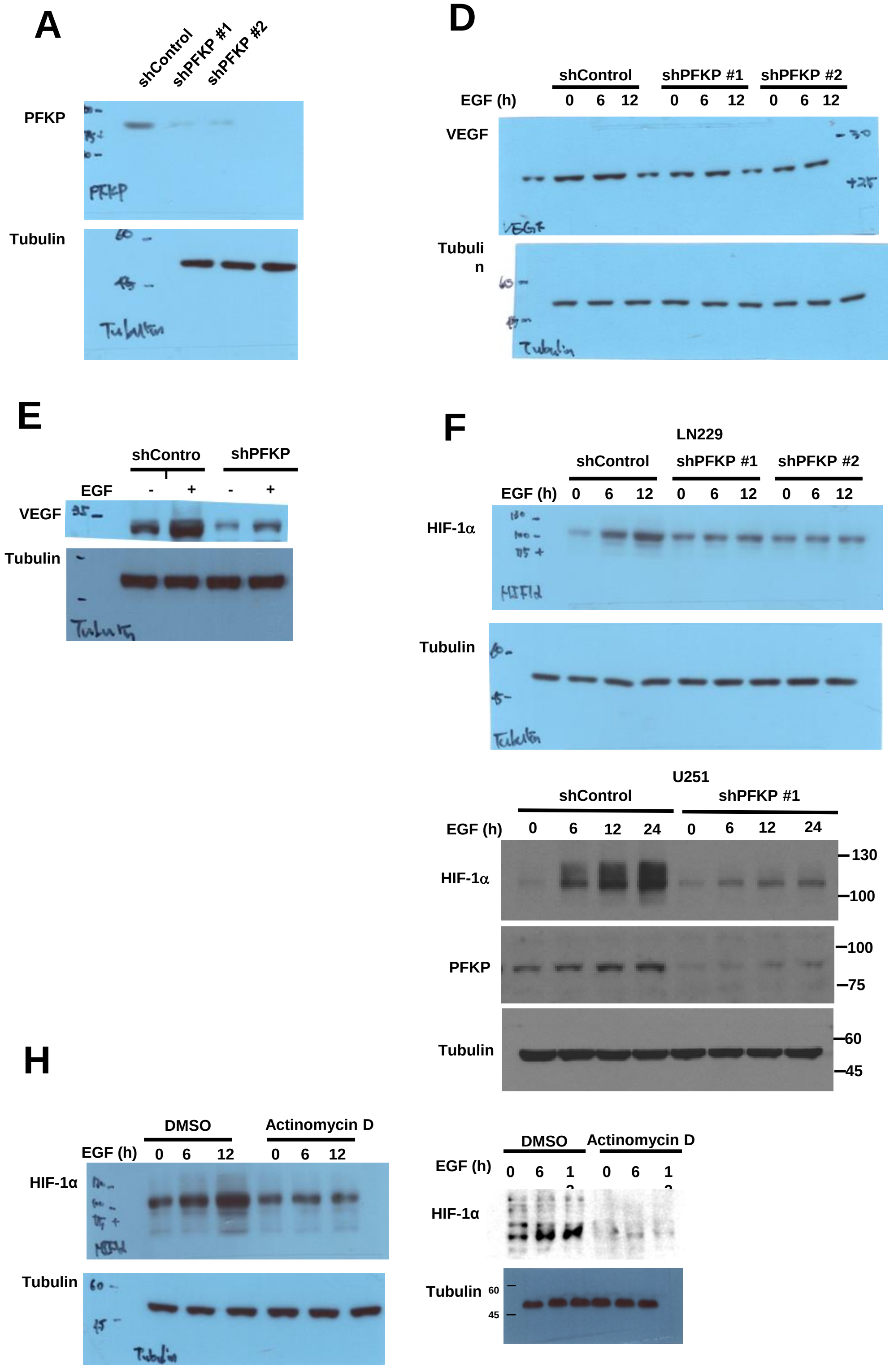
A
shControl
shPFKP #1
shPFKP #2
PFKP
Tubulin
D
shControl
shPFKP #1
shPFKP #2
EGF (h)
0 6 12 0 6 12 0 6 12
VEGF
Tubuli
n
E
shContro
l
shPFKP
EGF
- + - +
VEGF
Tubulin
F
LN229
shControl
shPFKP #1
shPFKP #2
EGF (h)
0 6 12 0 6 12 0 6 12
HIF-1α
Tubulin
U251
shControl
shPFKP #1
EGF (h)
0 6 12 24 0 6 12 24
HIF-1α
130
100
PFKP
100
75
Tubulin
60
45
H
DMSO
Actinomycin D
EGF (h)
0 6 12 0 6 12
HIF-1α
Tubulin
DMSO
Actinomycin D
EGF (h)
0 6 1
2
0 6 1
2
HIF-1α
Tubulin
60
45

Figure S2

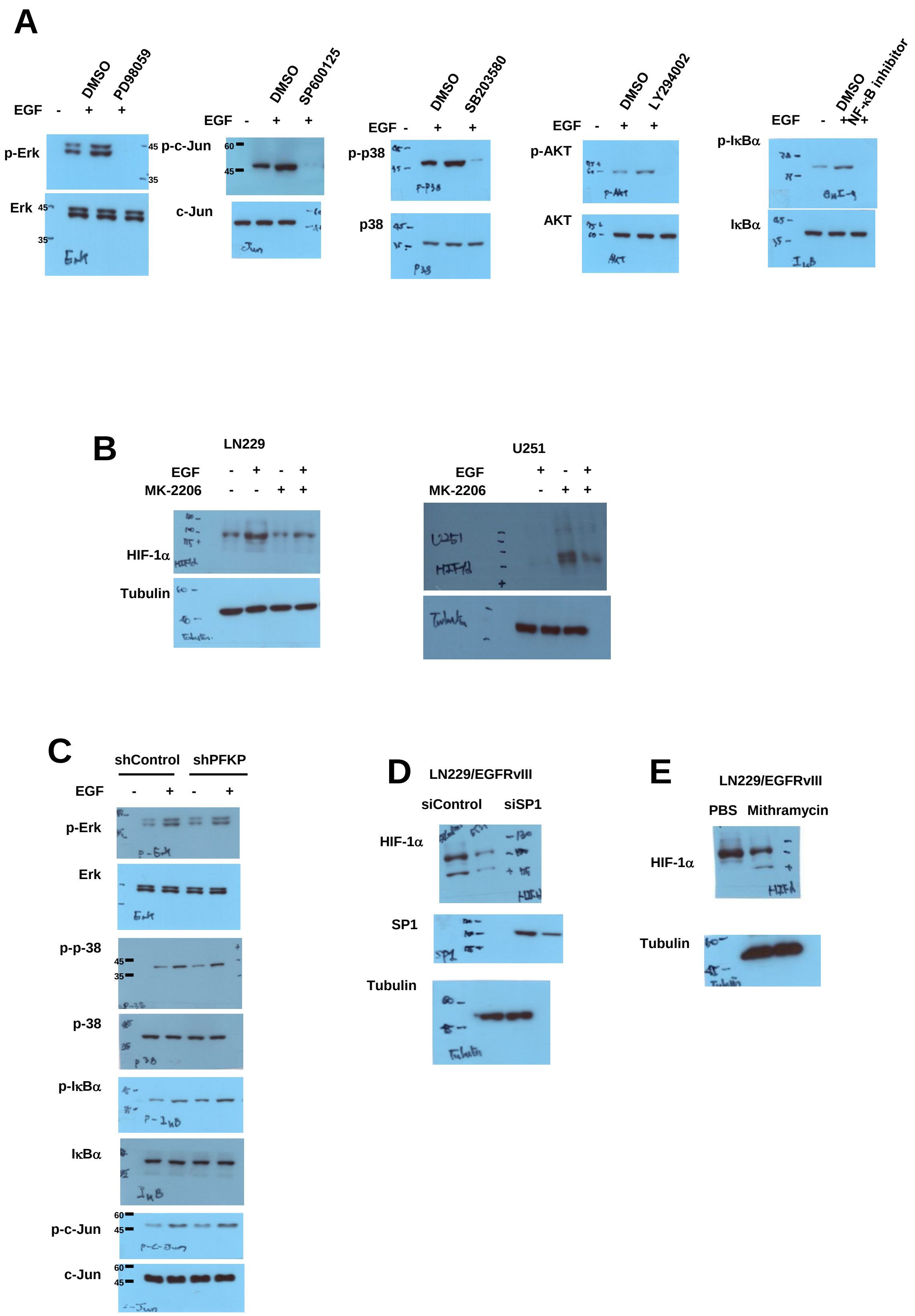

A
DMSO
PD98059
EGF - + +
p-Erk
Erk
45
35
SP600125
p-c-Jun
c-Jun
60
SB203580
p-p38
p38
LY294002
p-AKT
AKT
NF-κB inhibitor
p-IκBα
IκBα
B
LN229
U251
EGF
MK-2206
HIF-1α
Tubulin
C
shControl
shPFKP
p-p-38
p-38
D
LN229/EGFRvIII
siControl
siSP1
SP1
E
PBS
Mithramycin

Figure S3

A

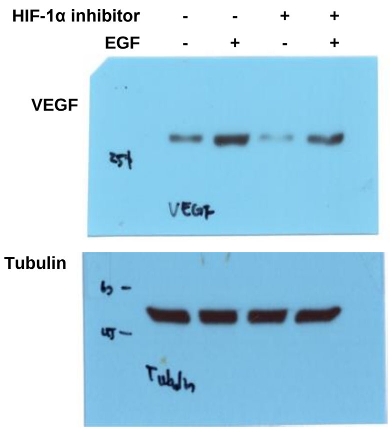

Figure S5

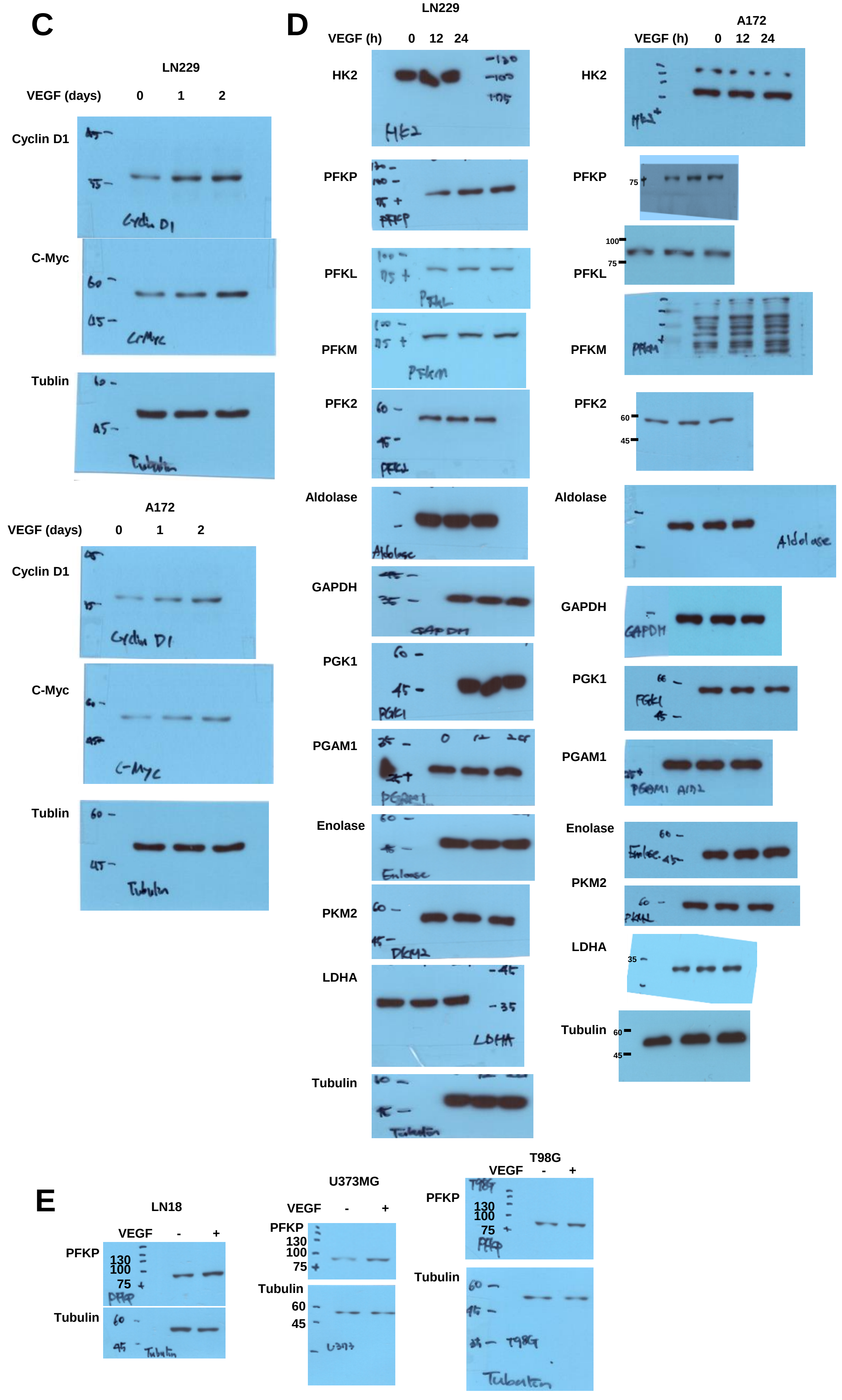
C
LN229
VEGF (days) 0 1 2
Cyclin D1
C-Myc
Tublin
A172
VEGF (days) 0 1 2
Cyclin D1
C-Myc
Tublin
D
LN229
VEGF (h) 0 12 24
HK2
PFKP
PFKL
PFKM
PFK2
Aldolase
GAPDH
PGK1
PGAM1
Enolase
PKM2
LDHA
Tubulin
A172
VEGF (h) 0 12 24
HK2
PFKP
75
100
75
PFKL
PFKM
PFK2
60
45
Aldolase
GAPDH
PGK1
PGAM1
Enolase
PKM2
LDHA
35
Tubulin
60
45
E
LN18
VEGF - +
PFKP
130
100
75
Tubulin
U373MG
VEGF - +
PFKP
130
100
75
Tubulin
60
45
T98G
VEGF - +
PFKP
130
100
75
Tubulin

Figure S5

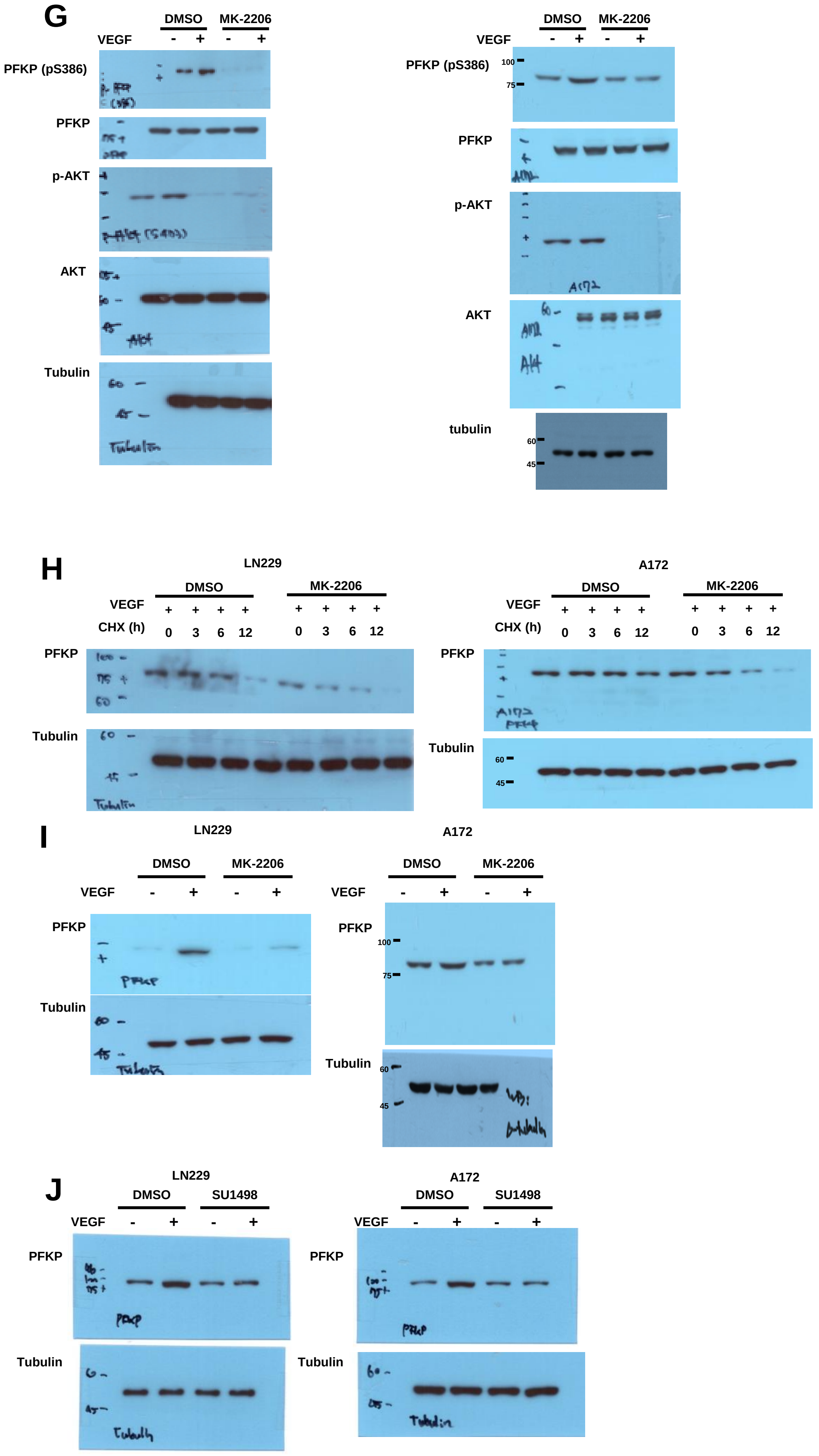
G
LN229
DMSO
MK-2206
VEGF
- + - +
PFKP (pS386)
PFKP
p-AKT
AKT
Tubulin
A172
DMSO
MK-2206
VEGF
- + - +
PFKP (pS386)
100
75
PFKP
p-AKT
AKT
tubulin
60
45
H
LN229
DMSO
MK-2206
VEGF
+ + + + + + + +
CHX (h)
0 3 6 12 0 3 6 12
PFKP
Tubulin
A172
DMSO
MK-2206
VEGF
+ + + + + + + +
CHX (h)
0 3 6 12 0 3 6 12
PFKP
Tubulin
60
45
I
LN229
DMSO
MK-2206
VEGF
- + - +
PFKP
Tubulin
A172
DMSO
MK-2206
VEGF
- + - +
PFKP
100
75
Tubulin
60
45
J
LN229
DMSO
SU1498
VEGF
- + - +
PFKP
Tubulin
A172
DMSO
SU1498
VEGF
- + - +
PFKP
Tubulin

Supplementary Figure legends

Supplementary Fig. 1 (related to Figure 1) PFKP depletion in GBM cells results in impaired EGFR activation-induced VEGF expression.

WB and qRT-PCR were performed with indicated primers and antibodies, respectively (**A, D-F, H**).

A LN229/EGFRvIII cells were transfected with shRNA against PFKP.

B Mouse survival times were recorded and visualized using Kaplan-Meier survival curves. Data represent the means ± SD of 7 mice.

C Representative H&E staining images of intracranial xenografts bearing LN229 cells stably expressing with or without PFKP shRNA (upper panel). IHC analyses of the tumor tissues with an anti-CD31 antibody and quantification of CD31 (bottom panel). Scale bar, 2 mm (upper panel) and 100 μm (bottom panel).

D Expression levels of VEGF in the LN229 cells stably expressing control shRNA or PFKP shRNA.

E Serum-starved U251 cells with or without PFKP depletion were treated with EGF (100 ng/mL) for 12 h.

F Serum-starved LN229 and U251 cells with or without PFKP depletion were treated with EGF (100 ng/mL).

G LN229/EGFRvIII were stably expressed with control shRNA or PFKP shRNA.

H Serum-starved LN229 and U251 cells were pretreated DMSO or actinomycin D (1 μg/mL) for

1 h and then stimulated with or without EGF (100 ng/mL).

I Serum-starved LN229 and U251 cells stably expressed control shRNA or shPFKP were treated with or without EGF (100 ng/mL) for 12 h.

Data are presented as mean ± standard deviation of three independent experiments (**D, E, G, I**). ***P < 0.001, based on the Student's t-test.

Supplementary Fig. 2 (related to Figure 2) PFKP Y64 phosphorylation induces EGFR activation-enhanced *HIF-1α* transcriptional expression through SP1 transactivation.

WB and qRT-PCR were performed with the indicated primers and antibodies, respectively (**A-E**).

A Serum-starved GSCs were pretreated with the indicated inhibitors for 1 h and then stimulated with or without EGF (100 ng/mL) for 30 min.

B Serum-starved LN229 and U251 cells were pretreated with DMSO or MK-2206 (5 μM) for 1h and then stimulated with or without EGF (100 ng/mL) for 12 h.

C Serum-starved GSCs stably expressing control shRNA or shPFKP were treated with or without EGF (100 ng/mL).

D LN229/EGFRvIII cells were transfected with control siRNA or SP1 siRNA.

E LN229/EGFRvIII cells were pretreated with PBS or mithramycin (500 nM) for 1 h and then stimulated with or without EGF (100 ng/mL).

Data are presented as mean ± standard deviation of three independent experiments (**B, D, E**). **P < 0.01; ***P < 0.001, based on the Student's t-test.

Supplementary Fig. 3 (related to Figure 3) PFKP Y64 phosphorylation induces VEGF expression through HIF-1 α expression and β-catenin Ser552 phosphorylation in response to EGFR activation.

A Serum-starved U251 cells were pre-treated DMSO or HIF-1α inhibitor (10 μM) for 1h and then stimulated with or without EGF (100 ng/mL) for 12 h. WB and qRT-PCR were performed with indicated primers and antibodies, respectively.

B Serum-starved LN229 and U251 cells were co-transfected with luciferase reporter plasmids (pGL3-HRE-luciferase) and the Renilla control plasmid. The cells were pretreated with DMSO or HIF-1α inhibitor (10 μM) for 1 h and then stimulated with or without EGF (100 ng/mL) for 12 h. Luciferase activity was measured.

Data are presented as mean ± standard deviation of three independent experiments. $^{**}P < 0.01$; $^{***}P < 0.001$, based on the Student's t-test.

Supplementary Fig. 4 (related to Figure 4) PFKP Y64 phosphorylation induces HIF-1 α expression, β-catenin S552 phosphorylation, and VEGF expression, and promotes blood vessel formation *in vivo*.

Mouse survival times were recorded and visualized using Kaplan-Meier survival curves. Data represent the means ± SD of 7 mice.

Supplementary Fig. 5 (related to Figure 5) VEGF induces PFKP expression, PFKP

expression, PFK enzyme activity, aerobic glycolysis, and proliferation in GBM cells

WB and qRT-PCR were performed with the indicated primers and antibodies, respectively (**C-E, G-J**).

A and **B** Serum-starved LN229 and A172 cells were treated with VEGF (20 ng/mL). Glucose consumption (**A**) and lactate secretion (**B**) were analyzed.

C LN229 and A172 cells in 0.1% serum medium were treated with VEGF (20 ng/mL) and then WST-8 assay was performed. WB was performed with indicated antibodies.

D and **F** Serum-starved LN229 and A172 cells were treated with VEGF (20 ng/mL). The indicated protein expression levels (**D**) and PFK enzymatic activity (**F**) were measured.

E Serum-starved LN18, U373MG, and T98G cells were treated with or without VEGF (20 ng/ml) for 12h.

G Serum-starved LN229 and A172 cells were pretreated with DMSO or MK-2206 (5 μM) for 1 h and then stimulated with VEGF (20 ng/mL) for 30 min.

H Serum-starved LN229 and A172 cells were pretreated with VEGF (20 ng/mL) for 1 h and then treated with cycloheximide (CHX;100 μg/mL) in the presence of DMSO or MK-2206 (5 μM). The quantification of PFKP levels relative to tubulin is shown (bottom panel).

I Serum-starved LN229 and A172 cells were pretreated with DMSO or MK-2206 (5 μM) for 1 h and then stimulated with or without VEGF (20 ng/mL) for 24 h.

J Serum-starved LN229 and A172 cells were pretreated with DMSO or SU1498 (30 μM) for 1 h and then stimulated with or without VEGF (20 ng/mL) for 24 h.

K-M A172 cells with or without the expression of PFKP shRNA and with or without the

reconstituted expression of WT Flag-rPFKP or Flag-rPFKP S386A were cultured in serum-free DMEM with or without VEGF (20 ng/mL) for 24 h. PFK enzymatic activity (**K**), glucose consumption **(L),** and lactate secretion **(M)** were analyzed.

N A172 cells with or without the expression of PFKP shRNA and with or without the reconstituted expression of WT Flag-rPFKP or Flag-rPFKP S386A were cultured in 0.1% serum medium with or without VEGF (20 ng/mL) and then WST-8 assay was performed.

Data are presented as mean ± standard deviation of three independent experiments (**A-C, F, H, K-N**). **P* < 0.05; ***P* < 0.01; ****P* < 0.001, based on the Student’s t-test or one-way ANOVA with Tukey’s post hoc test.
